# Identification of Antioxidant, Antimicrobial, and Cytotoxic Compounds From *Hymenocardia acida* Tul. Using Ultra‐High‐Performance Liquid Chromatography‐Quadrupole Exactive‐Orbitrap‐Mass Spectrometry and Molecular Networking Approach

**DOI:** 10.1002/cbdv.202502485

**Published:** 2025-12-17

**Authors:** Ntsoaki Joyce Malebo, Idah Tichaidza Manduna, Monizi Mawunu, Ramakwala Christinah Chokwe, Bafedile Tlhapi

**Affiliations:** ^1^ Center For Innovation in Learning and Teaching Central University of Technology Bloemfontein South Africa; ^2^ Center For Applied Food Sustainability and Biotechnology, Faculty of Health and Environmental Sciences Central University of Technology Bloemfontein South Africa; ^3^ Department of Agronomy Polytechnic Institute Kimpa Vita University Uíge Angola; ^4^ Department of Biology, Faculty of Science and Technology University of Kinshasa Kinshasa Democratic Republic of Congo; ^5^ Department of Chemistry, College of Science, Engineering and Technology University of South Africa Johannesburg South Africa

**Keywords:** antimicrobial activity, antioxidant activity, *Hymenocardia acida*, molecular networking, UHPLC‐Q Exactive‐Orbitrap‐MS

## Abstract

*Hymenocardia acida* Tul. is a medicinal plant used in Angola to treat microbial diseases. It is a well‐known plant among local populations; however, few studies have focused on the assessment of phytochemicals associated with their biological activities. Hence, there is a need to evaluate the metabolomic profile of compounds that contribute to the antimicrobial, antioxidant activities, and cytotoxic effects of *H. acida*. This study investigated the chemical profile, antimicrobial and antioxidant activities, and cytotoxic effects of compounds derived from different plant organs of *H. acida*. The compounds were analyzed using ultra‐high‐performance liquid chromatography‐quadrupole‐electrostatic field orbitrap mass spectrometry, and the resulting data were further analyzed using a molecular networking approach. Thirty‐two natural compounds were annotated, including flavonoids, fatty acids, coumarins, terpenoids, alkaloids, anthocyanins, saponins, polyphenols, glycosides, and disaccharides. These families of natural compounds were differentially distributed in various plant parts, indicating chemical differences among the leaf, root, and stem bark extracts of *H. acida*, which were annotated for the first time in this plant. The crude stem bark (IC_50_ = 3.031 ± 1.610 µg/mL) and root (IC_0.5_ = 0.019 ± 0.000 µg/mL) extracts exhibited the highest antioxidant activity, as determined by 2,2‐diphenyl‐1‐picrylhydrazyl radical scavenging and reducing power assays, respectively. In contrast, using microbroth dilution methods, the leaf extracts exhibited activity against all tested microorganisms (*Streptococcus agalactiae*, *Escherichia coli*, *Staphylococcus aureus*, *Candida tropicalis*, *Candida albicans*, *Staphylococcus* subsp. *aureus*, *Staphylococcus epidermidis*, *Klebsiella pneumonia*, and *Pseudomonas aeruginosa*) compared to the root and stem bark extracts, with minimal inhibitory concentrations ranging from 1 to 0.0078125 mg/mL. Stem bark and root extracts did not inhibit the visible growth of *E. coli*, *S. aureus*, *C. tropicalis*, and *C. albicans*. The methanol leaf extracts showed significant cytotoxic effects against Vero cells at 250 and 500 µg/mL, whereas the dichloromethane leaf extracts were cytotoxic to Vero cells at 500 µg/mL. The stem bark and root extracts were not cytotoxic at any concentration. Therefore, the annotated natural compounds may contribute to the antimicrobial, antioxidant, and cytotoxic effects of *H. acida*. These natural compounds offer promising avenues for the discovery and development of new lead drugs for the management and treatment of microbial diseases. Molecular networking provided a detailed phytochemical overview of this plant species. Furthermore, the results reported in this study highlight the importance of investigating bioactive chemical compounds in *H. acida* and provide new insights into the phytochemical annotation and pharmacological properties of extracts from various parts of *H. acida*.

## Graphical abstract

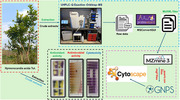

## 1 Introduction

*Hymenocardia acida* Tul. is an Angolan medicinal plant from the savannahs, locally known in Kikongo as Luvete, mpete, mvete, kihete, kiheta, and iheta [1, 2]. It is used in folk Angolan medicine to treat several conditions, such as anemia, bloody diarrhea, cough, hemorrhoids, lepra, inflammation of the legs, skin disease, open cervix, paralysis, weakness, worms, stimulation, muscular pain, menstrual pain, abdominal tumors, arthritis, headaches, hypertension, abscesses, toothaches, smallpox, diarrhea, rheumatic pain, chest complaints, and jaundice [1, 2, 3]. *H. acida* belongs to the family Phyllanthaceae, which is normally distributed in tropical Africa [4]. Research shows that this plant species is commonly used by many people in Angola and other African countries for the treatment of infections, microbial infections, and illnesses related to infections owing to its secondary metabolites [5, 6, 7]. Limited studies have demonstrated the antioxidant, antimicrobial, and cytotoxic capacity of the plant extracts from *H. acida* [4, 8, 9, 10]. However, despite being well known, the chemical profile of *H. acida* has not been well explained, and the compounds contributing to the biological activities of this plant species are unknown. Using a reductionist approach, studies on *H. acida* have shown very few isolated pure compounds, such as bisphenylpropanoid (katsumadin), a flavonolignan (hydnocarpin), a chromone (5,7‐dihydroxy‐2‐*n*‐pentacosanylchrom‐4‐one), a *C*‐glucosylated flavonoid (luteolin 6‐C‐β‐D‐glucopyranoside), a chromane stilbenoid (hymenocardichromanic acid), alkaloids (hymenocardine, hymenocardinol, hymenocardine *N*‐oxide and hymenocardine‐H), chromene stilbenoids (hymenocardichromene A, hymenocardichromene B, hymenocardichromene C, hymenocardichromene D, hymenocardichromene E and hymenocardichromene F), steroids (stigmasta‐3,5‐diene, β‐sitosterol and sitosterol‐3‐*O*‐β‐D‐glucopyranoside), and triterpenoids (friedelanone, betulinic acid, lupeol, and oleanolic acid) [10, 11]. Additionally, there is limited literature on the identification, annotation, characterization, and quantification of the compounds involved in the observed bioactivity of this plant. Therefore, it is necessary to determine the metabolites that contribute to the antioxidant, antimicrobial, and cytotoxic effects of *H. acida*. This study investigated the metabolomic profiles, in vitro antioxidant and antimicrobial potential, and cytotoxicity of secondary metabolites present in various plant organs of *H. acida* using ultra‐high‐performance liquid chromatography‐quadrupole‐electrostatic field orbitrap mass spectrometry (UHPLC‐Q Exactive‐Orbitrap‐MS) and molecular networking (MN) techniques. Moreover, it identified plant organs that may serve as a source of safe, bioactive compounds for managing microbial infections and infection‐related diseases using 2,2‐diphenyl‐1‐picrylhydrazyl (DPPH) free radical scavenging, reducing power, broth micro‐dilution, and 3‐(4,5‐dimethylthiazol‐2‐yl)‐2,5‐diphenyltetrazolium bromide (MTT) assays. These findings further revealed differences in the pharmacological activity of crude extracts from the aerial and underground parts of *H. acida*. These differences are likely attributable to a mixture of low‐molar‐mass constituents, which can influence their activities to varying degrees. These constituents include heterocyclic compounds, volatile compounds, and volatile oils. According to published studies, the composition of plant metabolites can vary significantly between various parts of the same plant when their pharmacological activity is being compared [12].

Metabolomics enables quantitative and qualitative analysis of a group of chemical compounds in a sample, whereby it classifies and quantifies complex mixtures of chemical compounds in a sample [13, 14]. The identification, annotation, characterization, and quantitation of compounds is supported through the application of spectrometric analytical methods such as LC/mass spectrometry (LC‐MS), gas chromatography/MS (GC‐MS), nuclear magnetic resonance (NMR) spectroscopy, and Fourier‐transform infrared spectroscopy (FTIR) [15,16]. Molecular networking is a tool used for visualizing, classifying, and identifying the structural link between metabolites belonging to the same chemical family, making it easier for researchers to characterize, annotate, and identify known metabolites in order to focus on unknown metabolites that might possibly be of biological interest [17]. A few investigations of the constituents in *H. acida* have employed proton NMR (^1^H‐NMR), carbon‐13 NMR (^13^C‐NMR), GC‐MS, and UHPLC‐Q Exactive‐Orbitrap‐MS analyses to characterize, identify, annotate, and quantify the constituents contributing to antioxidant and antibacterial activities [18, 19]. GC‐MS, LC‐MS, and UHPLC‐Q Exactive‐Orbitrap‐MS techniques can only tentatively suggest the identities of compounds, whereas the analysis of the NMR data of the isolated bioactive natural products provides the exact chemical structures of the isolated pure compounds. As previously stated, a few studies have shown that *H. acida* can be used to treat microbial infections; however, no studies have explored the phytochemistry of *H. acida* using a metabolomic approach and linked phytochemistry to pharmacological activities. Therefore, there is a need to apply these approaches or techniques to analyze, identify, and annotate possible antimicrobial compounds from various parts of *H. acida*. The resulting data were then correlated with plant antioxidant and antimicrobial activities, as well as cytotoxicity, which might potentially help pharmaceutical companies to further develop *H. acida* for the treatment and management of various diseases.

## 2 Results and Discussion

### 2.1 Metabolite Profiling Using UHPLC‐Q Exactive‐Orbitrap‐MS

Twenty‐six metabolites from different parts of *H. acida* were tentatively annotated using UHPLC‐Q/Orbitrap/MS analysis. The spectral data of the annotated metabolites were compared with values in the literature. UHPLC‐Q/Orbitrap/MS data for the annotated compounds, namely, retention time (Rt), molecular ions [M—H]^−^, MS/MS fragment ions (*m/z*, descriptions in Supporting Information), and main product ions, were provided in Table 1. The Rts of the metabolites were compared with the standard reference data to detect the metabolites in the *H. acida* extracts. Both electrospray ionization modes (ESI (‐/+); Figures S1 and S2) were generated using UHPLC‐Q/Orbitrap/MS. However, the negative ionization mode was chosen for further sample analysis because it produces a greater abundance of ions and provides information‐rich spectra. Various classes of secondary metabolites have been annotated, including flavonoids, fatty acids, coumarins, terpenoids, alkaloids, anthocyanins, saponins, polyphenols, and glycosides.

**TABLE 1:** Ultra‐high‐performance liquid chromatography‐quadrupole‐electrostatic field orbitrap mass spectrometry (UHPLC‐Q Exactive‐Orbitrap‐MS) data for compounds from various extracts of *H. acida*.

Compound number	Rt (min)	Theoretical mass [M‐H]^−^(*m/z*)	Observed mass [M‐H]^−^(*m/z*)	Molecular formula	MS/MS fragment ions (*m/z*)	Compound name	Compound class	Samples	References
**1**	1.52	339.12	339.20	C_20_H_20_O_5_	339	8‐Prenylnaringenin	Prenylated flavonoid	D,R,S	20
**2**	8.35	389.08	389.17	C_19_H_18_O_9_	389	Scaposin	Flavonoid	D,M,R,S	21
**3**	1.55	339.32	339.94	C_22_H_44_O_2_	337,319	Behenic acid	Saturated fatty acid	D	22
**4**	1.52	339.06	339.07	C_15_H_16_O_9_	338	6,7‐Dihydroxycoumarin‐6‐glucoside (Esculin)	Coumarin glucoside	D,R,S	23
**5**	1.50	483.31	483.12	C_30_H_44_O_5_	483	Poricoic acid B	Triterpenoid	D,M,R,S	24
**6**	0.65	427.18	427.18	C_23_H_28_N_2_O_6_	397,381,369	Isomajdine	Indole Alkaloid	D,M	25
**7**	0.23	339.17	339.12	C_20_H_24_N_2_O_3_	321,297	Yohimbic acid	Monoterpene indole alkaloids	D	26
**8**	1.96	251.07	251.03	C_16_H_12_O_3_	209,207	7‐Hydroxy‐3‐methylflavone	Flavonoid	D,M,R,S	27
**9**	1.76	596.17	596.16	[C_26_H_29_O_16_]+	595,593	Delphinidin‐3‐*O*‐sambubioside	Anthocyanins	M,R,S	28
**10**	1.68	595.13	595.15	C_26_H_28_O_16_	594,593	Quercetin‐3‐*O*‐vicianoside	Flavonoid	M	29
**11**	1.68	595.16	595.15	C_27_H_32_O_15_	595	Eriodictyol‐7‐*O*‐neohesperidoside (Neoeriocitrin)	Flavonoid	M,S	30
**12**	0.48	595.12	595.27	C_26_H_28_O_16_	595	Quercetin‐3‐arabinoglucoside (Peltatoside)	Flavonoid	M	31
**13**	1.45	919.45	919.50	C_45_H_74_O_19_	917,835	Furostane base ‐2H + 1O, O‐Hex, O‐Hex‐dHex	Saponin	R	MassBank‐RIKEN‐PR308985
**14**	4.84	1083.23	1083.25	C_51_H_86_O_24_	923,919,917	Furostane base + *O*‐Hex, *O*‐Hex‐Hex‐Hex	Saponin	R	MassBank‐RIKEN‐PR310726
**15**	3.83	467.35	467.16	C_31_H_48_O_3_	467	Dehydroeburicoic acid	Triterpenoid	S	32
**16**	2.58	295.17	295.23	C_16_H_25_NO_4_	—	Esmolol	Phenolic compound	R,S	33
**17**	2.88	297.23	297.24	C_17_H_14_O_5_	311	3',4'‐Dimethoxy‐7‐hydroxyflavone	Flavonoid	D,M,R,S	MassBank‐BS‐BS003750
**18**	1.46	293.17	293.18	C_17_H_26_O_4_	248,237,209	6‐Gingerol	Phenolic compound	D,M,R,S	33
**19**	1.55	501.32	501.11	C_30_H_46_O_6_	485,421	Medicagenic acid	Triterpenoids	R,S	34
**20**	2.33	277.18	277.20	C_17_H_26_O_3_	283,233,205	6‐Paradol	Phenolic ketone	D,M,R,S	33
**21**	1.97	447.09	447.09	C_21_H_20_O_11_	455,303	Quercitrin	Flavonoid	M,R,S	35
**22**	1.91	423.09	423.17	C_19_H_20_O_11_	313	3‐Glucosyl‐2,3′,4,4′,6‐ pentahydroxybenzophenone	Glycosides	D,M,R	33
**23**	1.46	533.12	533.38	C_24_H_22_O_13_	533	6ʹʹ ‐*O*‐Malonylgenistin	Glycosyloxy isoflavone	D,S	36
**24**	1.83	307.26	307.19	C_20_H_36_O_2_	290,289	Eicosadieneoic acid	Polyunsaturated fatty acid	D,M,R,S	37
**25**	1.98	525.16	525.30	C_23_H_28_O_11_	525	Paeoniflorin	Monoterpenoid glycoside	S	38
**26**	2.33	283.26	283.03	C_18_H_36_O_2_	283	Stearic acid	Saturated fatty acid	D,M,R,S	MSBNK‐Antwerp_Univ‐METOX_N109326_B8BB

*Note*: Qualitative annotation of compounds in *H. acida*: D—dichloromethane leaf extract; M—methanol leaf extract; R—root extract; and S—stem bark extract by UHPLC‐Q/Orbitrap/MS analysis.

Compound **1** was annotated with an [M‐H]^−^ ion at *m/z* 339.20 (Figure S3) as 8‐prenylnaringenin (C_20_H_20_O_5_) [20]. Compounds **2** (ion at *m/z*: 389.17, C_19_H_18_O_9_) and **3** (ion at *m/z*: 339.94, C_22_H_44_O_2_) were annotated as scaposin and behenic acid, respectively (Figures S4 and S5, respectively, in the Supporting Information). These compounds were previously reported by Khurm et al. and Isaac et al., respectively [21, 22]. Compound **4**, with a precursor ion at *m/z* 339.07 [M—H]^−^, was tentatively annotated as 6,7‐dihydroxycoumarin‐6‐glucoside (Esculin, Figure S6) [23]. Compound **5**, with a [M—H]^−^ ion at *m/z* 483.12 (Figure S7), was tentatively annotated as poricoic acid B with a molecular formula of C_30_H_44_O_5_ [24], whereas compound **6**, with a [M—H]^−^ ion at *m/z* 427.18 (Figure S8), was identified as isomajdine with a molecular formula of C_23_H_28_N_2_O_6_ [25]. Compounds **7** and **8** showed the presence of yohimbic acid (*m/z* 339.12, Figure S9) and 7‐hydroxy‐3‐methylflavone (*m/z* 251.03, Figure S10) [26, 27]. Furthermore, compounds **9** (Rt = 1.76 min), **10** (Rt = 1.68 min), **11** (Rt = 1.68 min), and **12** (Rt = 0.48 min), with [M—H]^−^ ions at *m/z* 596.16 (Figure S11), *m/z* 595.15 (Figure S12), *m/z* 595.15 (Figure S13), and *m/z* 595.27 (Figure S14), respectively were confirmed as delphinidin‐3‐*O*‐sambubioside ([C_26_H_29_O_16_]^+^), quercetin‐3‐*O*‐vicianoside (C_26_H_28_O_16_), eriodictyol‐7‐*O*‐neohesperidoside (Neoeriocitrin) (C_27_H_32_O_15_) and quercetin‐3‐arabinoglucoside (Peltatoside) (C_26_H_28_O_16_), respectively [28, 29, 30, 31]. Compounds **13** (*m/z* 919.50 [M—H]^−^, Figure S15), **14** (*m/z* 1083.25 [M—H]^−^, Figure S16), **15** (*m/z* 467.16 [M—H]^−^, Figure S17), and **16** (*m/z* 295.23 [M—H]^−^, Figure S18) were annotated as furostane base ‐1H2O + 1O, *O*‐Hex, *O*‐Hex‐Hex (C_45_H_74_O_19_), furostane base + *O*‐Hex, *O*‐Hex‐Hex‐Hex (C_51_H_86_O_24_), dehydroeburicoic acid (C_31_H_48_O_3_), and esmolol (C_16_H_25_NO_4_), respectively (MassBank‐RIKEN‐PR308985; MassBank‐RIKEN‐PR310726) [32, 33]. However, 3',4'‐dimethoxy‐7‐hydroxyflavone (**17**), 6‐gingerol (**18**), medicagenic acid (**19**), 6‐paradol (**20**), quercitrin (**21**), and 3‐glucosyl‐2,3′,4,4′,6‐ pentahydroxybenzophenone (**22**) with precursor ions at *m/z* 297.24 [M—H]^−^, *m/z* 293.18 [M—H]^−^, *m/z* 501.11 [M—H]^−^, *m/z* 277.20[M—H]^−^, *m/z* 447.09 [M—H]^−^ and *m/z* 423.17 [M—H]^−^, respectively were annotated at 2.88 min, 1.46 min, 1.55 min, 2.33 min, 1.97 min, and 1.91 min, respectively, as shown in Figures S19 and S20–S24, respectively in the Supporting Information) (MassBank‐BS‐BS003750) [33, 34, 35]. Compounds **23** (*m/z* 533.38 [M—H]^−^, Figure S25), **24** (*m/z* 307.19 [M—H]^−^, Figure S26), **25** (*m/z* 525.30 [M—H]^−^, Figure S27), and **26** (*m/z* 283.03 [M—H]^−^, Figure S28) were identified as 6ʹʹ ‐*O*‐malonylgenistin (C_24_H_22_O_13_), eicosadieneoic acid (C_20_H_36_O_2_), paeoniflorin (C_23_H_28_O_11_), and stearic acid (C_18_H_36_O_2_), respectively (MSBNK‐Antwerp_Univ METOX_N109326_B8BB) [36, 37, 38]. The UHPLC‐Q Exactive‐Orbitrap‐MS chemical profiles showed that various plant parts of *H. acida* produced both identical and non‐identical chemical molecules. Most of these phytochemicals were found in the leaf extracts compared to the root and stem bark extracts. To our knowledge, this is the first report of chemical molecules extracted from *H. acida plant parts*.

### 2.2 MN of *H. acida* Metabolites

The analysis of the different organs (root, leaf, and stem bark extracts) of *H. acida* was performed using the MN approach combined with UHPLC‐Q/Orbitrap/MS operating in negative mode (ESI^−^) as proposed on the Global Natural Product Social Molecular networking (GNPS) website: http://gnps.ucsd.edu (accessed on July 12, 2024). Spectral data were acquired using the data‐dependent scan (dd‐MS^2^) method, which creates second‐stage MS^2^ fragmentation data for each precursor ion (*m/z*) over a predetermined threshold. The UHPLC‐Q/Orbitrap/MS chromatograms of negative ESI (Figure S1) and positive ESI (Figure S2) of the *H. acida* crude extracts are provided in the Supporting Information. The chemical variety of the different plant organs of *H. acida* was investigated using the classical MN workflow on the GNPS platform, and the results were annotated. The generated molecular networks (Clusters **A**–**D**) were visualized in Cytoscape version 3.9.1, as shown in Figure 1 [39]. A total of 958 molecular ions were observed as nodes and 1403 as edges in the molecular network. The stem bark extracts had the highest number of nodes (297), followed by the root (257 nodes), methanol leaf (222 nodes), and dichloromethane leaf extracts (182 nodes). Furthermore, a combination of molecular network and manual confirmatory search allowed for the annotation of unique metabolites in the leaf, root, and stem bark extracts of *H. acida*. Cluster A (Figure 1) formed in MN was characterized by precursor ions with *m/z* 389.17, *m/z* 595.15, *m/z* 501.11, *m/z* 447.09, *m/z* 895.18 and *m/z* 432.09 which were annotated as scaposin (**2**), eriodictyol‐7‐*O*‐neohesperidoside (Neoeriocitrin) (**11**), medicagenic acid (**19**), quercitrin (**21**), orientin (**27**), and vitexin (**28**) respectively. In cluster B, the characteristic ions at *m/z* 295.23, 297.24, 293.18, and 231.15 were tentatively annotated as esmolol (**16**), 3', 4'‐dimethoxy‐7‐hydroxyflavone (**17**), 6‐gingerol (**18**), and costunolide (**29**), respectively. Moreover, cluster C, 7‐hydroxy‐3‐methylflavone (**8**), and 6‐gingerol (**18**), with precursor ions at *m/z* 251.03 and *m/z* 293.18, were present in all extracts. However, in cluster D, a metabolite at *m/z* 209.02, annotated as mucic acid (**30**), was present in the methanol leaf extract, whereas the metabolites at *m/z* 341.10 and *m/z* 387.11, annotated as sucrose (**31**) and alpha, alpha‐trehalose – 40.0 eV (**32**), were present in leaf and stem bark extracts.

FIGURE 1Molecular network of *Hymenocardia acida* crude extracts. Nodes of cluster A (phenylpropanoids and polyketides), cluster B (benzenoids), cluster C (lipids and lipid‐like molecules), and cluster D (organic oxygen compounds) were labeled with the precursor mass. The networking displayed as pie charts indicates the distribution of the ion intensities of the stem bark (blue), root (purple), methanol leaf (yellow), and dichloromethane leaf (green) crude extracts.

**Figure:**
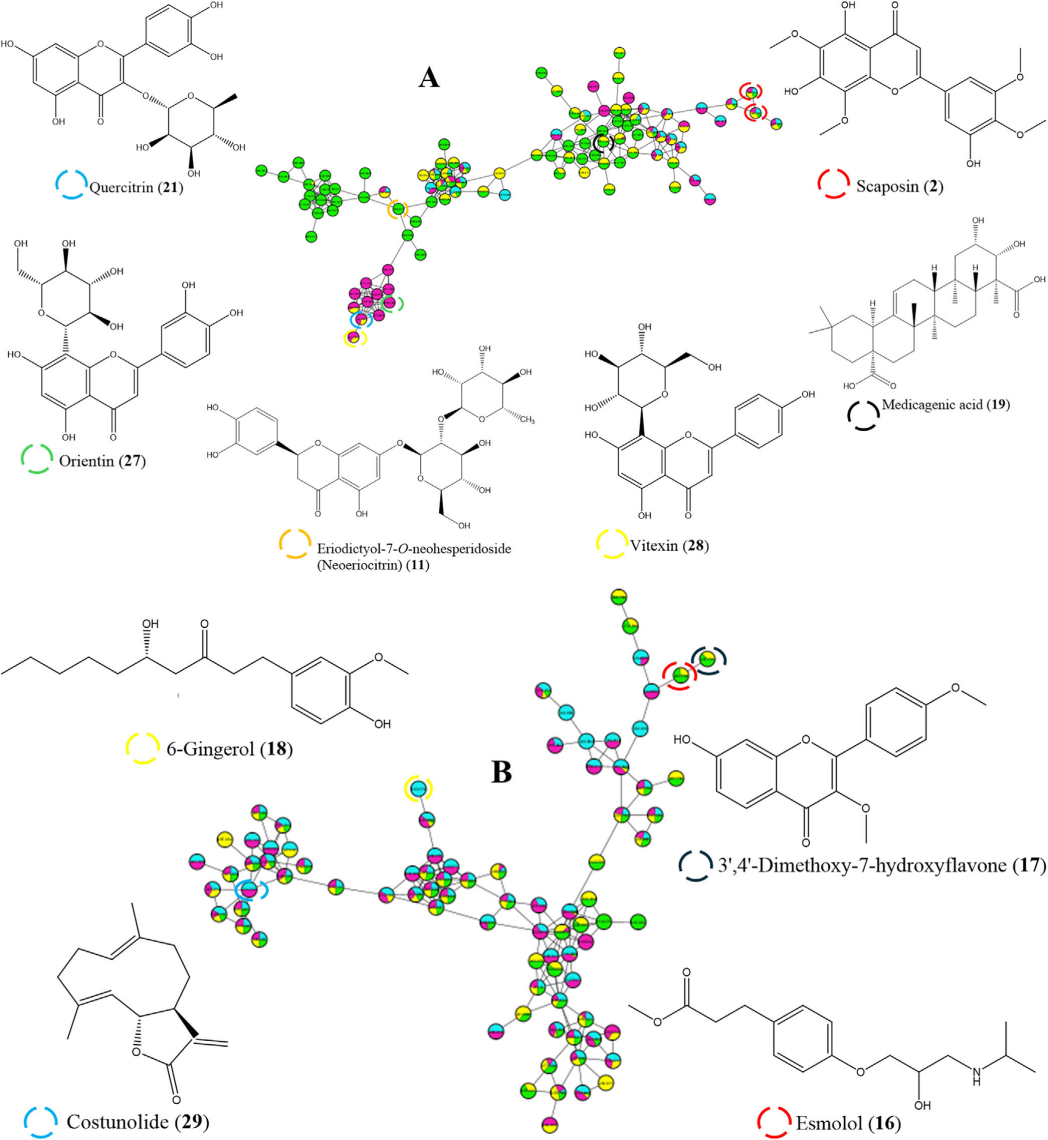

**Figure:**
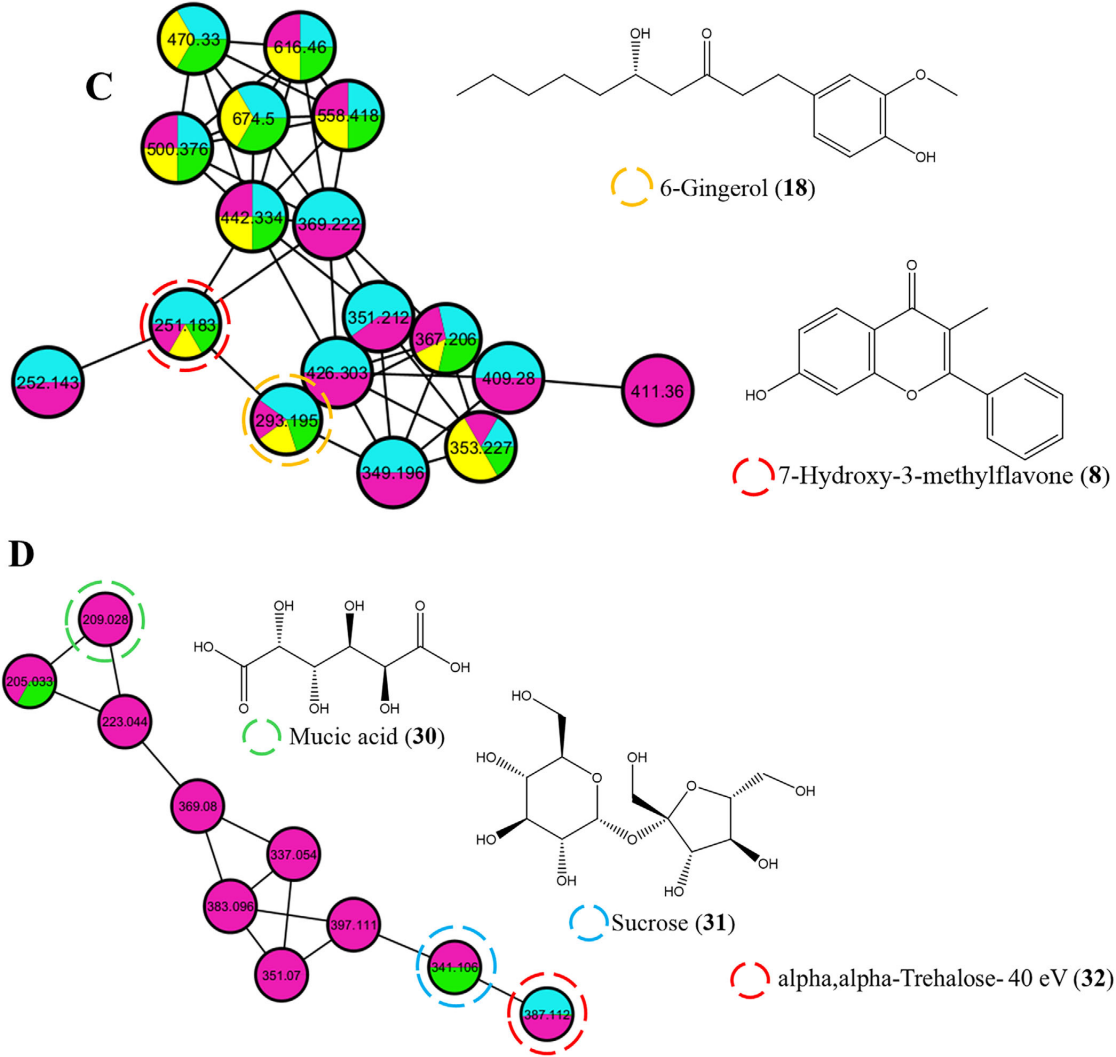

### 2.3 Antioxidant Activity of *H. acida* Extracts

The different extracts exhibited high DPPH radical scavenging and reducing power activities (Table 2). As shown in Table 2, the stem bark extract showed potent antioxidant activity, with an IC_50_ value of 3.031 ± 1.610 µg/mL using DPPH, whereas the root extract showed the lowest antioxidant activity, with an IC_50_ value of 27.232 ± 32.020 µg/mL. However, the root extracts showed potent reducing power activity with an IC_0.5_ value of 0.019 ± 0.000 µg/mL, whereas the stem bark extract had the lowest reducing power activity (IC_0.5_ = 0.045 ± 0.044 µg/mL). The presence of flavonoids, anthocyanins, monoterpenoid glycosides, and phenolic compounds tentatively identified in the stem bark and root extracts of *H. acida* (as shown in Table 2) might have affected the high antioxidant capacities of these crude extracts. Parthiban et al. reported that 7‐hydroxy‐3‐methylflavone (**8**) isolated from the leaves of *Avicennia officinalis* L. has antioxidant activity against DPPH with an IC_50_ value of 5.5486 ± 0.81 mg/mL [40]. On the other hand, Obouayeba et al. confirmed that the presence of delphinidin‐3‐*O*‐sambubioside (**19**) quantified using an HPLC analysis contributed to the antioxidant activity of the methanolic petals of Roselle (*Hibiscus sabdariffa* L.) using the DPPH radical scavenging assay [41]. Furthermore, Kim et al. proved that paeoniflorin (**25**), tentatively identified from the ethanol root extract of *Paeonia lactiflora* using HPLC analysis, contributed to the potent antioxidant activity of this plant species [42]. Che Zain et al. showed that orientin (**27**) and vitexin (**28**), identified using a UHPLC, were responsible for the antioxidant activity of the aqueous methanol leaves of *Elaeis guineensis* Jacq., as determined by DPPH and nitric oxide free radical scavenging assays (Figure 1, cluster A) [43]. In addition, Chen et al. and dos Santos et al. reported that 6‐gingerol (**18**) and quercitrin (**21**), detected using LC‐Q time‐of‐flight MS (LC‐QTOF‐MS), HPLC, and ESI‐MS/MS, contributed to the antioxidant activities of *Zingiber officinale* var. *roscoe* and *Erythrina speciosa* Andrews, as evidenced by the DPPH (1,1‐diphenyl2‐picrylhydrazyl) radical‐scavenging assay [44, 45]. Therefore, 7‐Hydroxy‐3‐methylflavone (**8**), delphinidin‐3‐*O*‐sambubioside (**9**), paeoniflorin (**25**), 6‐gingerol (**18**), and quercitrin (**21**) may be partly responsible for the antioxidant activity of the stem bark and roots extracts of *H. acida*. Other studies have shown that the antioxidant properties of several medicinal plant extracts are due to the high quality of flavonoids, terpenoids, and phenolic compounds [46]. The redox physical characteristics of phenolic compounds contribute to their antioxidant activity by acting as reducing agents, singlet oxygen quenchers, possible metal chelators, and hydrogen donors [47]. Furthermore, the hydroxyl groups (‐OH) in phenolics and their derivatives react with reactive oxygen species and reactive nitrogen species in a termination reaction, breaking the progression of new radical formation [47]. Zhou et al. reported that the different numbers of carbon–carbon unsaturated double bonds (C═C) in terpenoids are responsible for the strong antioxidant activities of these metabolites [48]. Furthermore, the outcomes of this study also showed that various crude extracts had greater antioxidant activities than the reference standards (ascorbic acid, gallic acid, and quercetin) using both DPPH radical scavenging and reducing power assays. This indicates that *H. acida* could be a useful antioxidant agent for the management of free radicals. To the best of our knowledge, there are no published reports or studies on the effects of phytochemicals on the antioxidant activities of different plant organs in *H. acida*. These findings provide valuable insights into the antioxidant potential of *H. acida*.

**TABLE 2:** Antioxidant activity of *Hymenocardia acida* extracts.

Samples	DPPH IC_50_ (µg/mL)	Reducing Power IC_0.5_ (µg/mL)
Root extract	27.232 ± 32.020^a^	0.019 ± 0.000^a^
Stem bark extract	3.031 ± 1.610^abc^	0.045 ± 0.044^a^
Methanol leaf extract	5.371 ± 0.556^a^	0.245 ± 0.208^a^
Dichloromethane leaf extract	9.198 ± 7.110^ab^	0.084 ± 0.044^a^
Ascorbic acid	44.308 ± 9.813^c^	0.355 ± 0.393^b^
Gallic acid	37.340 ± 17.529^abc^	8.210 ± 11.257^b^
Quercetin	37.361 ± 21.356^bc^	3.760 ± 2.979^b^

*Note*: Values with different superscript letters in each column are significantly different using one‐way analysis of variance (ANOVA) at *p* < 0.05. The data above are depicted by mean ± standard deviation of three replicates (*n* = 3). In the DPPH (2,2‐diphenyl‐1‐picrylhydrazyl) free radical scavenging activity: ^a^—Root extract was significantly different from ascorbic acid; ^abc^—Stem bark extract was not significantly different from all samples; ^ab^—Dichloromethane leaf extract was significantly different to ascorbic acid; ^c^—Ascorbic acid was significantly different from the root, methanol leaf, and dichloromethane leaf extracts; ^bc^—Quercetin was significantly different from the root and methanol leaf extracts. In the reducing power activity: ^a^—Root extract was significantly different from ascorbic acid, gallic acid, and quercetin; ^b^—Ascorbic acid was not significantly different from the root, stem bark, methanol leaf, and dichloromethane leaf extracts.

### 2.4 Antimicrobial Activity of *H. acida* Extracts

As shown in Table 3, the dichloromethane leaf, methanol leaf, stem bark, and root extracts exhibited greater activity against *Streptococcus agalactiae* and *Klebsiella pneumoniae*, with minimal inhibitory concentration (MIC) values ranging from 0.125 to 0.0078125 mg/mL. Our results are in agreement with the study conducted by Agbidye et al. (2020), who showed that root and stem bark extracts showed greater sensitivity against *Klebsiella pneumoniae*, with MIC values of 8.0 × 10^2^ and 10.0 × 10^2^ mg/mL [8]. A stronger inhibitory effect against *Candida tropicalis* and *Candida albicans* strains was presented by the dichloromethane and methanol leaf extracts, with MIC values of 0.125 and 0.0625 mg/mL, respectively, compared to the stem bark and root extracts, which did not inhibit the invisible growth of the microorganisms. The results obtained in this study do not support the results of a study conducted by Shimbe et al., who demonstrated that the root extract had higher antimicrobial activity against *Candida tropicalis* and *Candida albicans* strains with an MIC value of 0.62 µg/mL, respectively [4]. According to literature studies, several medicinal plants produce phytochemicals that exhibit pharmacological or toxicological activities [49, 50]. However, the appearance of various components and the growth of medicinal plants are influenced by environmental conditions, such as temperature, rainfall, light intensity, different soil characteristics, humidity, and changes in season. These environmental conditions should be taken into consideration for plant collection purposes to ensure a reproducible quantity of bioactive compounds [51]. In addition, the dichloromethane leaf extract (MIC = 0.125 mg/mL) demonstrated greater activity against *Staphylococcus epidermidis* compared to the methanol leaf, stem bark, and root extracts. The antimicrobial activity of the leaf, stem bark and root crude extracts may be due to the presence of costunolide (**29**), vitexin (**28**), orientin (**27**), 8‐prenylnaringenin (**1**), 6‐gingerol (**18**), medicagenic acid (**19**), 6‐paradol (**20**), paeoniflorin (**25**) annotated using UHPLC‐Q Exactive‐Orbitrap‐MS and MN approach (Table 1 and Figure 1). This is consistent with several studies that have shown the antimicrobial activity of these compounds against bacterial pathogens [52, 53, 54, 55, 56, 57, 58, 59, 60]. A study conducted by Koirala et al. and Mehmood et al. showed that flavonoids, phenolic compounds, and terpenoids play an important role in inhibiting bacterial growth [47, 61]. The presence of hydroxyl groups (‐OH) in the chemical structure of phenolics promotes interaction of phenolics by hydrogen‐binding with the microbial cell envelope [62]. Furthermore, the side chain attached at the C3 position of phenols reduces the level of oxidation that results in antimicrobial activity [47]. Monoterpenes and sesquiterpene oxygenated derivatives of terpenes are known for their ability to combat both bacteria susceptible to specific antibiotics at clinically achievable concentrations and antibiotic‐resistant strains [63]. In addition, these monoterpenes and sesquiterpene oxygenated derivatives of terpenes act primarily by disrupting microbial cell membranes, interfering with deoxyribonucleic acid replication, and inhibiting protein synthesis [63]. Therefore, 8‐prenylnaringenin (**1**), 6‐gingerol (**18**), medicagenic acid (**19**), 6‐paradol (**20**), paeoniflorin (**25**), orientin (**27**), vitexin (**28**), and costunolide (**29**) could be used as markers of the antimicrobial activities of *H. acida* Tul. Furthermore, the results of the current study showed that root extracts were more active than leaf and stem bark extracts against the selected bacterial strains. The outcomes of this study are in accordance with previous research studies conducted by Agbidye et al. and Shimbe et al., who revealed that the methanolic root crude extract of *H. acida* showed good antimicrobial activity against the tested strains [8, 4]. Presently, no literature studies exist that show which compounds are responsible for any antimicrobial activity of various plant parts of *H. acida* using a metabolomics approach. Overall, the findings of this study suggest that *H. acida* could be utilized as an active ingredient in antimicrobial products owing to its potential antimicrobial activity.

**TABLE 3:** Antimicrobial activity of *Hymenocardia acida* extracts.

Minimal inhibitory concentration (MIC) (mg/mL)									
Microbial species	*Streptococcus agalactiae*	*Escherichia coli*	*Staphylococcus aureus*	*Candida tropicalis*	*Candida albicans*	*Staphylococcus* subsp. *aureus*	*Staphylococcus epidermidis*	*Klebsiella pneumoniae*	*Pseudomonas aeruginosa*
Samples									
Dichloromethane leaf extract	0.03125	0.5	0.5	0.125	0.0625	0.25	0.125	0.015625	0.25
Methanol leaf extract	0.125	0.5	0.5	0.125	0.0625	1	1	0.0078125	0.5
Stem bark extract	0.125	—	—	—	—	1	1	0.0078125	0.5
Root extract	0.0625	—	—	—	—	0.5	1	0.0078125	0.25
Ampicillin	1	0.5	—	—	1	—	—	—	0.0078125
Chloramphenicol	1	0.0078125	—	—	0.0078125	0.25	0.125	—	0.015625
Ciprofloxacin	—	0.0078125	—	—	0.0078125	—	0.0078125	—	0.0078125
Erythromycin	0.0078125	0.125	—	—	0.0625	—	—	—	0.0078125
Gentamicin	—	—	—	—	0.0078125	—	—	0.0078125	—

*Note*: MIC of *Hymenocardia acida* extracts and antibiotic controls against selected bacterial pathogens tested in three replicates (*n* = 3).

### 2.5 Cytotoxicity of the Crude Extracts of *H. acida*

**FIGURE 2:**
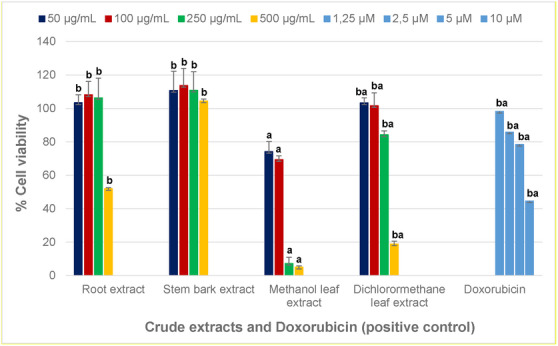
Cytotoxicity of *Hymenocardia acida* extracts against Vero cells at different concentrations of 50, 100, 250, and 500 µg/mL. Values with different superscript letters are significantly different according to one‐way ANOVA at *p* < 0.05. Data represent the mean ± standard deviation of two independent experiments. Data for doxorubicin (positive control) at concentrations of 1.25, 2.5, 5, and 10 µM are shown for comparison.

The cytotoxic effect of various parts of *H. acida* was determined using MTT against African green monkey (Vero) kidney cells (Figure 2). The inhibitory concentration (IC_50_, Table 4) was determined from the percentage of cell viability compared with that of doxorubicin (positive control, Table 4 and Figure 2). The root and stem bark extracts were not toxic at concentrations of 50, 100, 250, or 500 µg/mL (Figure 2). However, the methanol leaf extract was toxic at concentrations of 250 and 500 µg/mL but did not cause cytotoxicity at concentrations of 50 µg/mL and 100 µg/mL (Figure 2). Furthermore, dichloromethane leaf extract was not cytotoxic at concentrations of 50, 100, and 250 µg/mL; however was toxic at 250 µg/mL (Figure 2). This cytotoxicity may be due to the presence of delphinidin‐3‐*O*‐sambubioside (**9**, anthocyanin), isomajdine (**6**, alkaloid), and 6‐gingerol (**18**, phenolic compound) in the leaf extracts (Table 4). This is consistent with several studies that have shown the cytotoxicity of these compounds against African green monkey (Vero) kidney cells [59, 64, 65]. It should be considered that the in vitro results are not necessarily indicative of what could happen in vivo; therefore, the leaf extracts should be investigated in an in vivo model at varying concentrations to confirm the toxicity of their phytoconstituents. In addition, the cytotoxicity of *H. acida* in Vero cells has not been determined before. To our knowledge, this is the first study on the cytotoxicity of different plant organs of *H. acida* against these cells.

**TABLE 4:** Cytotoxic effects of *Hymenocardia acida* extracts and doxorubicin against African green monkey (Vero) kidney cells.

Samples	Vero IC_50_ (µg/mL)
Root extract	362.30 ± 2.33^c^
Stem bark extract	117.40 ± 2.50^b^
Methanol leaf extract	>500
Dichloromethane leaf extract	>500
Doxorubicin (µM; positive control)	9.198 ± 7.110^a^

*Note*: Values above are presented by mean ± standard deviation of three replicates (*n* = 3). Values with different superscript letters in each column are significantly different using one‐way ANOVA at *p < 0.05*.

### 2.6 Summary

In summary, this study investigated the chemical profile, antioxidant, and antimicrobial activities, as well as the cytotoxic effects of compounds from different plant organs of *H. acida* Tul. A wide range of phytochemical compounds was tentatively annotated from the stem bark, root, and leaf extracts of *H. acida* using UHPLC‐Q Exactive‐Orbitrap‐MS and MN approaches. Most of these phytochemical compounds were annotated in the leaf extracts. Chemical profiling revealed that the stem bark, root, and leaf extracts of *H. acida* produced similar and different phytochemical compounds. These findings demonstrate that metabolomics offers notable advantages over classical techniques, which rely on the isolation and purification of natural products that are time‐consuming, tedious, expensive, and inefficient in the investigation of phytoconstituents. The stem bark and root extracts exhibited strong antioxidant activity, whereas the leaf extracts showed potent antimicrobial activity against all tested microorganisms. Based on the findings of this study, *H. acida* is a highly recommended source of potent antioxidant and antimicrobial compounds. This study showed that the stem bark and root extracts of *H. acida* were not cytotoxic to African green monkey (Vero) kidney cells at varying concentrations. Therefore, the safe and effective phytochemical compounds in these crude extracts may be potential leads for the treatment and management of various diseases. Collectively, these results highlight that *H. acida* is a promising source of phytochemical compounds that could be used to develop novel drugs for the treatment and management of infectious diseases. Further isolation and purification of antioxidant and antimicrobial compounds from *H. acida* are required to discover other bioactive compounds that contribute to the biological activities of this plant species.

## 3 Conclusions

In this study, antimicrobial and antioxidant activities, including the cytotoxicity of *H. acida*, were investigated using a combined UHPLC‐Q Exactive‐Orbitrap‐MS and MN approach. The UHPLC‐Q Exactive‐Orbitrap‐MS and MN approaches were used to annotate thirty‐two metabolites. This study demonstrated that MN is an effective tool for investigating non‐targeted mass spectrometry data. Future perspectives will involve further mining of these molecules using improved computational tools to analyze complex molecular structures, ultimately leading to a more profound understanding of plant chemistry and prospective healing applications. Furthermore, the stem bark and root extracts showed higher antioxidant activities than the leaf extracts. The study further showed that leaf extracts exhibited antimicrobial activity against all tested bacterial strains compared to stem and root extracts. The results of this study also indicate that certain parts of *H. acida* exhibit cytotoxic effects against Vero cells. It is important to note that in vitro cytotoxicity outcomes serve as a preliminary screening tool to recognize or identify potentially safe or toxic plant extracts or metabolites and do not ultimately report general toxicity. Furthermore, it should be noted that in vitro outcomes are not indicative of in vivo outcomes. Therefore, the screened plant extracts or metabolites should be placed in an in vivo model to assess their toxicity. In addition, the findings of this study comprehensively indicate that anthocyanins, alkaloids, phenolic compounds, flavonoids, terpenoids, fatty acids, coumarins, and glycosides contribute to antimicrobial and antioxidant activities, as well as cytotoxicity. This study is the first to determine the metabolites responsible for the antimicrobial and antioxidant activities, as well as the cytotoxicity of *H. acida*, using a UHPLC‐Q Exactive‐Orbitrap‐MS and MN approach.

## 4 Experimental

### 4.1 General Experimental Procedure

All solvents and chemicals used in this study were of analytical grade. Trypan blue, trypsin/EDTA, penicillin/streptomycin, L‐glutamine, fetal bovine serum, Earle's balanced salt solution, and minimal essential medium were obtained from Cytiva HyClone (Marlborough, MA, USA). African green monkey (Vero) kidney cells (ATCC CCL‐81, RRID: CVCL_0059) were purchased from Cytiva HyClone (Marlborough, MA, USA). Gentamicin and iodonitrotetrazolium (INT) were purchased from Mchem Lab Suppliers (Cape Town, South Africa). Acetonitrile, methanol, dichloromethane, and formic acid were purchased from Sigma‐Aldrich (St. Louis, MO, USA). Mueller–Hinton agar I, Mueller–Hinton broth, gallic acid, quercetin, ascorbic acid, dimethyl sulfoxide, DPPH, ampicillin, erythromycin, chloramphenicol, and ciprofloxacin were purchased from Sigma‐Aldrich (Darmstadt, Germany).

### 4.2 Plant Collection, Sampling, and Extraction

Fresh plant samples were collected from Angola (“Latitude: S 7°37′1.54812” “Longitude: E 14°58′28.85988”) on March 14, 2023. Botanical identification was confirmed by Mr. Monizi Mawunu of the Department of Agronomy, Polytechnic Institute, Kimpa Vita University, Angola. An herbarium reference specimen (UNIKIVI‐012‐2023), was deposited at the Department of Agronomy, Polytechnic Institute, Kimpa Vita University (Angola). The samples were air‐dried for four weeks and then ground to a fine powder using a POLYMIX lab mill with blade grinding, PX‐MFC 90 D (Willows, Bloemfontein, South Africa). Grounded root (approximately 65.05 g) and stem bark (approximately 240.95 g) samples of *H. acida* were macerated in 2 L of methanol (100%) at room temperature for 48 h, followed by filtration. Büchi Rotavapor (Sigma‐Aldrich, St. Louis, MO, USA) was used to concentrate the root (1.95 g) and stem bark (16.48 g) extracts. In addition, ground leaves (approximately 225.62 g) were macerated in 2 L of dichloromethane (100%) and methanol (100%) for 48 h at room temperature, followed by filtration. Büchi Rotavapor (Sigma‐Aldrich, St. Louis, MO, USA) was used to concentrate the dichloromethane (4.79 g) and methanol (16.29 g) leaf extracts.

### 4.3 UHPLC‐Q Exactive‐Orbitrap‐MS Analysis

The extracts were annotated using a Q Exactive Plus Orbitrap mass spectrometer coupled with a Thermo Scientific Dionex Ultimate 3000 UHPLC system (Thermo Fisher Scientific, Waltham, MA, USA). Exactive Plus was coupled to the MS with a heated ESI probe and an optimum source (capillary temperature, 290°C; sheath gas flow, 50 arbitrary units; spray voltage, 3 kV; auxiliary temperature, 400°C). Full mass spectrometry selected ion monitoring and data‐dependent MS^2^ (dd‐MS^2^) with both positive and negative polarity switching over a scan range of *m/z* 100–1500 with a mass tolerance window of ˂5 ppm was used for the two analysis modes. The mass spectrometer was operated at a resolution of 70 000 full width at half maximum in full scan mode, with an automatic gain control target set at 1.0 × 10^6^ and a maximum injection time of 100 ms. LC‐ESI‐Orbitrap‐MS analysis was performed using a C18 analytical column (4.6 x 150 mm, particle size 3.5 µm) to separate the extracts. The mobile phases used were 0.1% (v/v) formic acid in water (solvent A) and 0.1% (v/v) formic acid in acetonitrile (solvent B). Linear gradient elution was initiated with 5% solvent B at 0 min and then increased to 100% solvent B for 20 min. The mobile phase flow rate was 0.9 mL min^−1^, the sample injection volume was 10 µL, and the column temperature was 25°C. Data processing was performed using XCaliber version 3.0 software (Thermo Fisher Scientific Inc., Waltham, MA, USA). Both the negative (Figure S1) and positive (Figure S2) ESI modes were evaluated; however, the negative mode was selected for further sample analysis because it resulted in a greater abundance of ions and provided information‐rich spectra. Moreover, the mass spectrometry raw data files were converted to an open‐source format (.mzXML) using the ProteoWizard tool MSConvertGUI (version 3.0.24164‐38d6037) software. The mzXML files were evaluated using MZmine version 3.9.0; centroid techniques, such as mass detection, chromatograph building, and peak deconvolution, were used to process the MZmine version 3.9.0 settings. The aligned peak list, which contained the Rt, *m/z* values, and peak heights in each sample, was exported in the ‐quant.csv format. The exported file was uploaded to Mass Bank, an online database for compound identification. Phytochemicals with a match of ≥ 80% in the database were considered, and the identified compounds were extracted from the sample chromatogram data for further validation. A data‐dependent scan (dd‐MS^2^) was used to acquire high‐quality MS^2^ data. The top five most intense precursors were automatically selected for MS/MS fragmentation using an HCD. The parameters of dd‐MS^2^ were as follows: resolution, 17,500; AGC target, 1 × 10^5^; Maximum IT, 50 ms; loop count, 5; isolation window, 4 *m/z*. The Normalized Collision Energy was set to 20, 40, and 60 V.

### 4.4 MN Analysis

The analysis of the *H. acida* crude extracts was performed using the MN approach, as proposed on the Global Natural Product Social Molecular networking website: (http://gnps.ucsd.edu, accessed on July 12 2024). The mass spectroscopic data were transformed into the mzXML format using the open‐source ProteoWizard tool MSConvertGUI software (version 3.0.24164‐38d6037), and the spectral data were uploaded to WinSCP, which can be used to transfer files or data online. Once the spectral files or data were uploaded, MN linked the mass spectra of the compounds based on the similarity of their MS/MS fragment ions. The generated spectral network was imported using the Cytoscape (Version 3.91) software to visualize the network [39]. For compound annotations, the empirical formulas generated from the accurate mass obtained from the MS/MS data of the compounds were used to annotate the matched and some unmatched nodes, and these were compared to other common natural product dereplication databases, such as the Dictionary of Natural Products (http://dnp.chemnetbase.com/faces/chemical/ChemicalSearch.xhtml (accessed on August 07 2024)), ChemSpider (www.chemspider.com (accessed on September 05 2024) and KNApSAck (www.knapsackfamily.com (accessed on October 02 2024)).

### 4.5 Antioxidant Assay

#### 4.5.1 DPPH Free Radical Scavenging Activity Assay

The DPPH free radical scavenging activity was determined following the method described by Tlhapi et al. [66]. One hundred microliters of the crude extracts were added in triplicate into the first three wells of a 96‐well plate containing 100 µL of distilled water. Two hundred microliters of 0.3 M DPPH/methanol solution was added to all the wells. The plates were incubated at room temperature for 30 min, and the absorbance was measured on a microplate reader (SoftMax^R^ Pro 6 (version 6.3)) at a wavelength of 517 nm.

DPPH inhibitory percentage was calculated using the following formula: (Equation (1)).

(1) $$\mathrm{DPPH}\;\mathrm{RSA}\;\left(\%\right)=\frac{\mathrm{ADPPH}\;-\mathrm{Asample}}{\mathrm{ADPPH}\;}\;\times 100$$

A_DPPH_ = absorbance of DPPH solution.

A_sample_ = absorbance of the extracts, reference standards, and DPPH.

*Notes*: The ability of the crude extracts and reference standards to inhibit fifty percent of the free radicals (IC_50_) was extrapolated from a graph of % RSA against concentration.

#### 4.5.2 Reducing Power Assay

The reducing power of the crude extracts was estimated as described by a slight modification of the method by Tlhapi et al. [66]. Fifty microliters of the crude extracts were mixed with sodium phosphate buffer (0.2 M, pH 6.6; 50 µL) and 50 µL of 1% aqueous potassium hexacyanoferrate [K_3_Fe (CN)_6_] solution. After 20 min of incubation at 50°C, 50 µL of 10% trichloroacetic acid solution and 80 µL of each mixture were transferred to a 96‐well plate containing 80 µL of distilled water and ferric chloride (0.1%, w/v; 16 µL). Absorbance was measured on a microplate reader (SoftMax^R^ Pro 6 (version 6.3)) at a wavelength of 700 nm. All crude extracts and reference standards (ascorbic acid, gallic acid, and quercetin) were tested in triplicate.

### 4.6 Antimicrobial Assay

#### 4.6.1 Test Microorganisms

*Escherichia coli* (ATCC 25922); *Staphylococcus aureus* (ATCC 25923); *Staphylococcus* subsp. *aureus* (ATCC 43300); *Staphylococcus epidermidis* (ATCC 12223); *Klebsiella pneumoniae* (ATCC 27736); *Pseudomonas aeruginosa* (ATCC 27853) and *Streptococcus agalactiae* strains; and yeast isolates (*Candida tropicalis* and *Candida albicans*) used in this study were obtained from the Centre for Applied Food Sustainability and Biotechnology (CAFSaB), Central University of Technology (Bloemfontein campus). The organisms were sub‐cultured in a petri dish containing Mueller–Hinton agar I and incubated at 37°C for 24 h. Before testing, a 0.5 McFarland standard was prepared and used in the microdilution assay.

#### 4.6.2 Determination of the MIC

The MIC was evaluated using the microbroth dilution method in sterile 96‐well plates as described by Ngobeni et al. [67]. One hundred microliters of sterilized Mueller–Hinton broth was added to all the wells of a 96‐well plate, followed by the addition of 100 µL of the crude extracts to all the wells of the first row. A two‐fold serial dilution was performed to create a concentration sequence ranging from 1 to 0.0078125 mg/mL. After serial dilutions of the extracts and antibiotic controls (ampicillin, gentamicin, erythromycin, chloramphenicol, and ciprofloxacin), 100 µL of 0.5 McFarland bacterial suspensions (1.5x10^8^ CFU/mL) were added to all wells, and the plates were incubated at 37°C for 24 h. After incubation, 40 µL of 0.4 mg/mL INT was added to each well, and the results were read by observing the color change. The MIC was recorded as the lowest concentration of the crude extract that inhibited visible growth. Ampicillin, gentamicin, erythromycin, chloramphenicol, and ciprofloxacin, standard antibacterial drugs, were used as positive controls. A minimum of three repetitions was performed for each plant extract and strain.

### 4.7 Cytotoxicity Testing

#### 4.7.1 Cell Culture

African green monkey (Vero) kidney cells (ATCC CCL‐81, RRID: CVCL_0059; Cellonex, South Africa) were cultured in minimal essential medium containing Earle's balanced salt solution and L‐glutamine (2.0 mM) (Cytiva HyClone, Marlborough, MA, USA), supplemented with 10% fetal bovine serum and 1% penicillin/streptomycin (P/S; Biowest, Nuaille, France). The cells were maintained at 37°C with 95% air and 5% CO_2_ in an incubator (Nϋve, Ankara, Turkey). Thereafter, the cells were trypsinized with 0.25% trypsin/EDTA (Cytiva HyClone, Marlborough, MA, USA) and split at a ratio of 1:5 for further passaging at 70%–80% confluency. Trypan blue 0.4% (Cytiva Hyclone, Marlborough, MA, USA) on an automated cell counter (NanoEntek, Guro‐gu, Seoul, South Korea) was used to determine cell viability, and only suspensions with a cell viability higher than 90% were used for the cytotoxicity assay.

#### 4.7.2 Cytotoxicity Assay

The cytotoxicity of crude extracts was determined using the MTT assay described by Mariri et al. [68]. The African green monkey kidney (Vero) cells were seeded (10 000 cells per well) in 96‐well microliter plates and treated with doxorubicin (positive control; Merck, Darmstadt, Germany) and crude extracts at different concentrations. The cells were allowed to attach overnight under standard cell‐culture conditions. The cells were initially treated with different concentrations of plant extracts (500, 250, 100, and 50 µg/mL) dissolved in dimethyl sulfoxide and further diluted in fresh culture medium. In each test, the concentration of dimethyl sulfoxide (negative control) in the medium did not exceed 0.5%. Doxorubicin (Merck, Darmstadt, Germany) at 10, 5, 2.5, and 1.25 µM was used as a positive control. After further incubation of the plates for 48 h under standard cell‐culture conditions, the culture medium containing the tested samples was removed from the plates and substituted with fresh medium (200 µL) with 30 µL of thiazolyl blue tetrazolium bromide (5 mg/mL) (Sigma‐Aldrich, St. Louis, Missouri, USA) dissolved in phosphate‐buffered saline. After 4 h at 37°C with 5% CO_2_, the culture medium was aspirated using a suction pump (Integra Biosciences Corp., USA), and 50 µL of dimethyl sulfoxide was added to all wells. The absorbance was measured using a SpectraMax iD3 multimode microplate reader (Winooski, VT, USA) at a wavelength of 570 nm. Cell viability was calculated as a percentage of the negative control (DMSO 0.5%) (See Formula 2).

The crude extracts were calculated using the following formula:

(2) $$\mathrm{Cell}\;\mathrm{viability}\;\left(\%\right)=\frac{\mathrm{Abs}\;\left(\mathrm{sample}\right)\;-Abs\;\left(Blank\right)}{\mathrm{Abs}\;\left(\mathrm{negative}\;\mathrm{control}\right)\;-Abs\;\left(Blank\right)}\;\times 100$$

Abs = absorbance.

### 4.8 Statistical Analysis

Antioxidant, antimicrobial, and cytotoxicity tests were conducted in triplicate. For antioxidant activity and cytotoxicity, the data are presented as the mean ± standard deviation. Ascorbic acid, gallic acid, and quercetin were used as reference standards for the antioxidant tests, and doxorubicin was used as a positive control for the cytotoxicity test. Ampicillin, gentamicin, erythromycin, chloramphenicol, and ciprofloxacin, standard antibacterial drugs, were used as positive controls for the antimicrobial test. One‐way analysis of variance with Duncan's Multiple Range Test was used to test differences in the measured variables between extracts in the antioxidant and cytotoxicity tests. Statistical analysis was conducted using the IBM SPSS Statistics package, version 22 (Chicago, IL, USA); *p* < 0.05 was considered statistically significant in all tests.

## Author Contributions

**Ntsoaki Joyce Malebo**: conceptualized and revised the manuscript. **Idah Tichaidza Manduna**: conceptualized and revised the manuscript. **Monizi Mawunu**: conceptualized and revised the manuscript. **Ramakwala Christinah Chokwe**: performed UHPLC–Q/Orbitrap/MS analyses and revised the manuscript. **Bafedile Tlhapi**: conceptualization, performed the experiments and biological assays, analyzed the data, wrote the manuscript, and revised the manuscript. All authors have read and agreed to the published version of the manuscript.

## Conflicts of Interest

The authors declare no conflicts of interest.

## Supporting information




**Supporting File 1**: cbdv70802‐sup‐0001‐SuppMat.docx.

## Data Availability

The data that support the findings of this study are available from the corresponding author upon reasonable request.

## References

[1] A. Göhre , Á. B. Toto‐Nienguesse , M. Futuro , C. Neinhuis , and T. Lautenschläger , “Plants From Disturbed Savannah Vegetation and Their Usage by Bakongo Tribes in Uíge, Northern Angola,” Journal of Ethnobiology and Ethnomedicine 12 (2016): 42, 10.1186/s13002-016-0116-9. 27650466 PMC5030725

[2] T. Lautenschläger , M. Monizi , M. Pedro , et al., “First Large‐scale Ethnobotanical Survey in the Province of Uíge, northern Angola,” Journal of Ethnobiology and Ethnomedicine 14 (2018): 51, 10.1186/s13002-018-0238-3. 30045744 PMC6060550

[3] D. Tlhapi , N. Malebo , I. T. Manduna , T. Lautenschläger , and M. Mawunu , “A Review of Medicinal Plants Used in the Management of Microbial Infections in Angola,” Plants 13 (2024): 2991, 10.3390/plants13212991. 39519911 PMC11548206

[4] R. Y. Shimbe , T. A. Tor‐Anyiin , M. E. Khan , and J. V. Anyam , “Î^2^‐Sitosterol From *Hymenocardia acida* Root Extract and Its Antimicrobial Activity",” Journal Of Chemical Society Of Nigeria 41 (2016): 76–81.

[5] S. Danladi , N. B. Lawal , A. M. Alhassan , et al., “In‐silico Investigation of the Antischizophrenic Activity of Phytochemical Constituents of *Hymenocardia acida* Tul. (Phyllantaceae) ",” Tropical Journal of Natural Product Research 9 (2025): 1309, 10.26538/tjnpr/v9i3.55.

[6] O. Adedokun , E. N. Ntungwe , C. Viegas , et al., “Enhanced Anticancer Activity of *Hymenocardia acida* Stem Bark Extract Loaded Into PLGA Nanoparticles",” Pharmaceuticals 15 (2022): 535, 10.3390/ph15050535. 35631361 PMC9147688

[7] A. M. Usman , N. M. Danjuma , J. Ya'u , et al., “Antidiarrhoeal Potentials of Methanol Bark Extract of *Hymenocardia acida* Tul. (Euphorbiaceae) in Laboratory Animals",” Bulletin of the National Research Centre 45 (2021): 118, 10.1186/s42269-021-00575-1.

[8] I. G. Agbidye , A. Q. Msughter , N. J. Chinelo , and S. D. Iortyom , “Phytochemical Screening and Antimicrobial Analysis of *Hymenocardia acida* ,” Chemical Research Journal 5 (2020): 81–93.

[9] S. Danladi , N. B. Lawal , and A. M. Alhassan , “Review on the Phytochemical and Pharmacological Activities of *Hymenocardia acida* Tul. (Phyllantaceae),” Journal of Current Biomedical Research 1 (2021): 92–105.

[10] A. R. D. Nanfack , A. A. S. Metiave , F. L. M. Dongmo , et al., “Secondary Metabolites From the Leaves of *Hymenocardia acida* and Their Chemotaxonomic Significance",” Biochemical Systematics and Ecology 113 (2024): 104799, 10.1016/j.bse.2024.104799.

[11] E. Tuenter , V. Exarchou , A. Baldé , et al., “Cyclopeptide Alkaloids From *Hymenocardia acida* ,” Journal of Natural Products 79 (2016): 1746–1751, 10.1021/acs.jnatprod.6b00131. 27351950

[12] D. Tlhapi , I. Ramaite , C. Anokwuru , and T. van Ree , “Molecular Networking‐Based Metabolome, in Vitro Antidiabetic and Anti‐Inflammatory Effects of *Breonadia Salicina* (Vahl) Hepper & J.R.I. Wood,” Metabolites 14 (2024): 291, 10.3390/metabo14060291. 38921427 PMC11206052

[13] Y. Liang , X. Ke , Z. Xiao , et al., “Untargeted Metabolomic Profiling Using UHPLC‐QTOF/MS Reveals Metabolic Alterations Associated With Autism,” BioMed Research International 2020 (2020): 6105608, 10.1155/2020/6105608. 32964039 PMC7502129

[14] C. J. Seneviratne , T. Suriyanarayanan , A. S. Widyarman , et al., “Multi‐omics Tools for Studying Microbial Biofilms: Current Perspectives and Future Directions,” Critical Reviews in Microbiology 46 (2020): 759–778, 10.1080/1040841X.2020.1828817. 33030973

[15] X. Li , P. Wang , Y. Tong , J. Liu , and G. Shu , “UHPLC‐Q‐Exactive Orbitrap MS/MS‐based Untargeted Metabolomics and Molecular Networking Reveal the Differential Chemical Constituents of the Bulbs and Flowers of *Fritillaria thunbergii* ,” Molecules 27 (2022): 6944, 10.3390/molecules27206944. 36296537 PMC9609367

[16] S. Z. Abd Ghafar , S. Muthukrishnan , N. K. Z. Zolkeflee , I. Natrah , and F. Abas , “Identification of Metabolites From *Halamphora* Sp. and Its Correlation With Quorum Sensing Inhibitory Activity via UHPLC‐ESI‐MS/MS‐Based Metabolomics and Molecular Networking,” Chemistry & Biodiversity 22 (2025): e20240228, 10.1002/cbdv.202402282. 39617725

[17] N. Mad Nasir , N. S. Ezam Shah , N. Z. Zainal , N. K. Kassim , S. M. M. Faudzi , and H. Hasan , “Combination of Molecular Networking and LC‐MS/MS Profiling in Investigating the Interrelationships between the Antioxidant and Antimicrobial Properties of *Curculigo latifolia* ,” Plants 10 (2021): 1488, 10.3390/plants10081488. 34451533 PMC8401502

[18] O. E. Yakubu , R. N. Boyi , C. Shaibu , M. A. Abah , and J. Akighir , “Antioxidant Parameters and GC‐MS Phytochemical Analysis of *Hymenocardia acida* Stem Bark Ethanolic Extract",” Trends in Applied Science Research 14 (2019): 263–270, 10.3923/tasr.2019.263.270.

[19] C. M. Starks , R. B. Williams , V. L. Norman , et al., “Antibacterial Chromene and Chromane Stilbenoids From *Hymenocardia acida* ,” Phytochemistry 98 (2014): 216–222, 10.1016/j.phytochem.2013.11.012. 24361290

[20] S. van Dinteren , C. Araya‐Cloutier , W. J. de Bruijn , and J. P. Vincken , “A Targeted Prenylation Analysis by a Combination of IT‐MS and HR‐MS: Identification of Prenyl Number, Configuration, and Position in Different Subclasses of (Iso) Flavonoids,” Analytica Chimica Acta (2021): 338874, 10.1016/j.aca.2021.338874. 34538332

[21] M. Khurm , Y. Guo , Q. Wu , et al., “ *Conocarpus lancifolius* (Combretaceae): Pharmacological Effects, LC‐ESI‐MS/MS Profiling and in Silico Attributes,” Metabolites 13 (2023): 794, 10.3390/metabo13070794. 37512501 PMC10385132

[22] J. G. de Oliveira Neto , J. L. Simplício , M. V. S. Junior , et al., “A Multifaceted Study on the Polymorphic Phase C of Behenic Acid Crystal: Structure, Physicochemical Characterization, Computational and Microbiological Insights,” Journal of Molecular Structure 2025 (1348): 143392, 10.1016/j.molstruc.2025.143392.

[23] C. K. Smitha and P. S. Udayan , “GC‐MS and HR‐LCMS Fingerprinting of Various Parts of *Oroxylum indicum* (L.) Vent. A Comparative Phytochemical Study Based on Plant Part Substitution Approach,” Journal of Pharmacognosy and Phytochemistry 9 (2020): 1817–1824.

[24] Y. Desmiaty , F. C. Saputri , M. Hanafi , R. Prastiwi , and B. Elya , “Anti‐elastase, Anti‐tyrosinase and Anti‐oxidant of *Rubus fraxinifolius* Stem Methanolic Extract,” Pharmacognosy Journal 12 (2020): 271–275, 10.5530/pj.2020.12.42.

[25] M. Picardo , J. Sanchís , O. Núñez , and M. Farré , “Suspect Screening of Natural Toxins in Surface and Drinking Water by High Performance Liquid Chromatography and High‐resolution Mass Spectrometry,” Chemosphere 261 (2020): 127888, 10.1016/j.chemosphere.2020.127888. 33113669

[26] N. A. Rahim , M. N. F. Roslan , M. Muhamad , and A. Seeni , “Antioxidant Activity, Total Phenolic and Flavonoid Content and LC–MS Profiling of Leaves Extracts of *Alstonia angustiloba* ,” Separations 9 (2022): 234, 10.3390/separations9090234.

[27] C. Bhajan , J. G. Soulange , V. M. R. Sanmukhiya , R. Olędzki , and J. Harasym , “Phytochemical Composition and Antioxidant Properties of *Tambourissa Ficus*, a Mauritian Endemic Fruit,” Applied Sciences 13 (2023): 10908, 10.3390/app131910908.

[28] K. Li , L. Liu , P. Xiong , et al., “Rapid Identification of Anthocyanin From the Epicarp of *Kadsura coccinea* (Lem.) AC Smith by UHPLC‐Q‐Exactive Orbitrap Mass Spectrometry",” Food Analytical Methods 14 (2021): 2545–2555, 10.1007/s12161-021-02038-9.

[29] T. Iwashina , R. Yangzom , H. P. Devkota , and T. Mizuno , “Flavonoids From the Leaves and Flowers of the Himalayan *Cathcartia villosa* (Papaveraceae)",” Biochemical Systematics and Ecology 96 (2021): 104267, 10.1016/j.bse.2021.104267.

[30] F. T. Abdl Aziz , A. S. Temraz , and M. A. Hassan , “Metabolites Profiling by LC‐ESI‐MS/MS Technique and in‐Vitro Antioxidant Activity of *Bauhinia madagascariensis* Desv. And *Bauhinia purpurea* L. Aerial Parts Cultivated in Egypt: A Comparative Study,” Azhar International Journal of Pharmaceutical and Medical Sciences 4 (2024): 169–188, 10.21608/aijpms.2023.212409.1215.

[31] Y. Kim , D. J. Lim , J. S. Song , J. A. Kim , B. H. Lee , and Y. K. Son , “Identification and Comparison of Bioactive Components of Two *Dryopteris* sp. Extract Using LC‐QTOF‐MS,” Plants 11 (2022): 3233, 10.3390/plants11233233. 36501275 PMC9740439

[32] M. Yang , Y. Zhao , Y. Qin , R. Xu , Z. Yang , and H. Peng , “Untargeted Metabolomics and Targeted Quantitative Analysis of Temporal and Spatial Variations in Specialized Metabolites Accumulation in *Poria cocos* (Schw.) Wolf (Fushen)",” Frontiers in Plant Science 12 (2021): 713490, 10.3389/fpls.2021.713490. 34621284 PMC8490877

[33] N. M. Peixoto Araujo , H. S. Arruda , F. N. Dos Santos , D. R. De Morais , G. A. Pereira , and G. M. Pastore , “LC‐MS/MS Screening and Identification of Bioactive Compounds in Leaves, Pulp and Seed From *Eugenia calycina* Cambess,” Food Research International 137 (2020): 109556, 10.1016/j.foodres.2020.109556. 33233178

[34] D. Li , D. Liu , M. Lv , P. Gao , and X. Liu , “Isolation of Triterpenoid Saponins From *Medicago sativa* L. With Neuroprotective Activities,” Bioorganic and Medicinal Chemistry Letters 30 (2020): 126956, 10.1016/j.bmcl.2020.126956. 31932222

[35] A. L. Santos , M. G. Soares , L. S. de Medeiros , M. J. Ferreira , and P. Sartorelli , “Identification of Flavonoid‐3‐*O*‐glycosides From Leaves of *Casearia arborea* (Salicaceae) by UHPLC‐DAD‐ESI‐HRMS/MS Combined With Molecular Networking and NMR",” Phytochemical Analysis 32 (2021): 891–898, 10.1002/pca.3032. 33554403

[36] H. S. Kiani , W. Ahmad , S. Nawaz , M. A. Farah , and A. Ali , “Optimized Extraction of Polyphenols From Unconventional Edible Plants: LC‐MS/MS Profiling of Polyphenols, Biological Functions, Molecular Docking, and Pharmacokinetics Study,” Molecules 28 (2023): 6703, 10.3390/molecules28186703. 37764478 PMC10534510

[37] H. Servi , Ö. Kisa , A. I. Aysal , G. Erköse Genç , and D. Şatana , “Chemical Profile by LC‐Q‐TOF‐MS of *Nigella sativa* Seed Extracts and in Vitro Antimicrobial Activity on Bacteria Which Are Determined Resistance Gene and Isolated From Nosocomial Infection",” Journal of Research in Pharmacy 26 (2022): 287, 10.29228/jrp.127.

[38] M. Takayama , M. Ubukata , K. Nagatomo , J. Tamura , and A. Kubota , “Quantum Chemical Analysis of Molecular and Fragment Ions Produced by Field Ionization of Methyl Stearate,” Journal of the American Society for Mass Spectrometry 34 (2023): 2731–2738, 10.1021/jasms.3c00277. 37902792

[39] P. Spohr , K. Dinkla , G. W. Klau , and M. El‐Kebir , “eXamine: Visualizing Annotated Networks in Cytoscape,” F1000Research 7 (2018): 519, 10.12688/f1000research.14612.2. 29983924 PMC6013758

[40] A. Parthiban , V. Sachithanandam , P. Lalitha , et al., “Isolation and Biological Evaluation 7‐hydroxy Flavone From Avicennia officinalis L: Insights From Extensive in Vitro, DFT, Molecular Docking and Molecular Dynamics Simulation Studies,” Journal of Biomolecular Structure and Dynamics 41 (2023): 2848–2860, 10.1080/07391102.2022.2039771. 35193476

[41] A. P. Obouayeba , N. B. Djyh , S. Diabate , et al., “Phytochemical and Antioxidant Activity of Roselle (*Hibiscus sabdariffa* L.) Petal Extracts",” Research Journal of Pharmaceutical, Biological and Chemical Sciences 5 (2014): 1453–1465.

[42] M. J. Kim , H. H. Kang , Y. J. Seo , K. M. Kim , Y. J. Kim , and S. K. Jung , “ *Paeonia Lactiflora* Root Extract and Its Components Reduce Biomarkers of Early Atherosclerosis via Anti‐inflammatory and Antioxidant Effects in Vitro and in Vivo",” Antioxidants 10 (2021): 1507, 10.3390/antiox10101507. 34679642 PMC8532938

[43] M. S. Che Zain , S. Y. Lee , C. Y. Teo , and K. Shaari , “Adsorption/Desorption Characteristics and Simultaneous Enrichment of Orientin, Isoorientin, Vitexin and Isovitexin From Hydrolyzed Oil Palm Leaf Extract Using Macroporous Resins,” Processes 9 (2021): 659, 10.3390/pr9040659.

[44] M. Chen , E. Lin , R. Xiao , Z. Li , B. Liu , and J. Wang , “Structural Characteristic, Strong Antioxidant, and Anti‐gastric Cancer Investigations on an Oleoresin From Ginger (*Zingiber officinale* var. *roscoe*),” Foods 13 (2024): 1498, 10.3390/foods13101498. 38790798 PMC11119446

[45] A. E. dos Santos , N. K. Simas , and R. M. Kuster , “Phytochemical Profiling, Antioxidant, and Phytotoxic Potentials of *Erythrina Speciosa* Andrews Leaves,” Ciência e Natura 46 (2024): e86537, 10.5902/2179460X86537.

[46] O. S. Nwozo , E. M. Effiong , P. M. Aja , and C. G. Awuchi , “Antioxidant, Phytochemical, and Therapeutic Properties of Medicinal Plants: A Review,” International Journal of Food Properties 26 (2023): 359–388, 10.1080/10942912.2022.2157425.

[47] A. Mehmood , S. Javid , M. F. Khan , K. S. Ahmad , and A. Mustafa , “In Vitro Total Phenolics, Total Flavonoids, Antioxidant and Antibacterial Activities of Selected Medicinal Plants Using Different Solvent Systems,” BMC Chemistry 16 (2022): 64, 10.1186/s13065-022-00858-2. 36030245 PMC9419333

[48] X. Zhou , T. Zheng , Y. Xie , et al., “Astaxanthin Accumulation in *Microcystis aeruginosa* under Different Light Quality",” Bioresource Technology 346 (2022): 126629, 10.1016/j.biortech.2021.126629. 34974094

[49] L. Tariq , B. A. Bhat , S. S. Hamdani , and R. A. Mir , “Phytochemistry, Pharmacology and Toxicity of Medicinal Plants,” in Medicinal and Aromatic Plants: Healthcare and Industrial Applications, eds. T. Aftab and K. R. Hakeem (Springer International Publishing, 2021), 217–240, 10.1007/978-3-030-58975-2_8.

[50] D. Tlhapi , I. Ramaite , C. Anokwuru , T. van Ree , N. Madala , and H. Hoppe , “Effects of Seasonal Variation on Phytochemicals Contributing to the Antimalarial and Antitrypanosomal Activities of *Breonadia salicina* Using a Metabolomic Approach",” Heliyon 10 (2024): e24068, 10.1016/j.heliyon.2024.e24068. 38298618 PMC10827688

[51] N. A. C. De Souza , P. G. S. D. Sá , T. C. D. L. Araújo , et al., “Seasonal Effect on the Chemical Composition of Mimosa Tenuiflora (Willd.) Poir Stem Bark From the Semi‐Arid Region of Pernambuco, Brazil,” Chemistry and Biodiversity 0 (2025): e02234, 10.1002/cbdv.202502234. 40986385

[52] M. F. Soliman , Y. M. Shetaia , A. A. Tayel , et al., “Exploring the Antifungal Activity and Action of *Saussurea costus* Root Extracts Against *Candida albicans* and Non‐albicans Species",” Antibiotics 11 (2022): 327, 10.3390/antibiotics11030327. 35326790 PMC8944531

[53] G. Ozbey , M. N. Muz , E. S. Tanriverdi , et al., “Chemical Composition, Antimicrobial Activities, and Molecular Docking Studies of Turkish Propolis Ethanol Extract,” Czech Journal of Food Science 41 (2023): 144–154, 10.17221/100/2022-CJFS.

[54] M. A. Daga , T. S. Ayala , and R. A. Menolli , “A Review of the Anti‐inflammatory and Antimicrobial Activities of the Components of the *Cecropia* Genus",” Asian Journal of Pharmaceutical and Clinical Research 13 (2020): 13–20, 10.22159/ajpcr.2020.v13i8.38031.

[55] D. Cao , Z. Zhang , X. Jiang , et al., “ *Psoralea corylifolia* L. and Its Active Component Isobavachalcone Demonstrate Antibacterial Activity Against *Mycobacterium abscessus* ,” Journal of Ethnopharmacology 329 (2024): 118142, 10.1016/j.jep.2024.118142. 38583730

[56] M. A. Elfaky , H. M. Okairy , H. M. Abdallah , et al., “Assessing the Antibacterial Potential of 6‐Gingerol: Combined Experimental and Computational Approaches,” Saudi Pharmaceutical Journal 32 (2024): 102041, 10.1016/j.jsps.2024.102041. 38558886 PMC10981156

[57] D. Charalambous , M. Christoforou , E. N. Kitiri , et al., “Antimicrobial Activities of *Saponaria cypria* Boiss. Root Extracts, and the Identification of Nine Saponins and Six Phenolic Compounds,” Molecules 27 (2022): 5812, 10.3390/molecules27185812. 36144548 PMC9505039

[58] H. M. Okairy , A. E. Koshak , M. A. Elfaky , et al., “6‐Paradol Exhibits Antimicrobial, Anti‐quorum Sensing and Anti‐Virulence Capacities on Gram‐negative Bacteria: In Vitro and in Vivo Studies,” South African Journal of Botany 174 (2024): 694–701, 10.1016/j.sajb.2024.09.034.

[59] W. Qian , J. Zhang , W. Wang , et al., “Antimicrobial and Antibiofilm Activities of Paeoniflorin Against Carbapenem‐Resistant Klebsiella pneumoniae,” Journal of Applied Microbiology 128 (2020): 401–413, 10.1111/jam.14480. 31602708

[60] Y. R. Choi and M. K. Kang , “Evaluation of Cytotoxic and Antibacterial Effect of Methanolic Extract of *Paeonia lactiflora* ,” Medicina 58 (2022): 1272, 10.3390/medicina58091272. 36143949 PMC9505222

[61] N. Koirala , C. Dhakal , N. N. Munankarmi , et al., “ *Vitex negundo* Linn.: Phytochemical Composition, Nutritional Analysis, and Antioxidant and Antimicrobial Activity,” Cellular and Molecular Biology 66 (2020): 1–7, 10.14715/cmb/2020.66.4.1. 32583767

[62] N. Oulahal and P. Degraeve , “Phenolic‐rich Plant Extracts With Antimicrobial Activity: An Alternative to Food Preservatives and Biocides?” Frontiers in Microbiology 12 (2022): 753518, 10.3389/fmicb.2021.753518. 35058892 PMC8764166

[63] S. Khanam , P. Mishra , T. Faruqui , et al., “Plant‐based Secondary Metabolites as Natural Remedies: A Comprehensive Review on Terpenes and Their Therapeutic Applications,” Frontiers in Pharmacology 16 (2025): 1587215, 10.3389/fphar.2025.1587215. 40458805 PMC12127327

[64] B. Hamrita , N. Emira , A. Papetti , et al., “Phytochemical Analysis, Antioxidant, Antimicrobial, and Anti‐Swarming Properties of *Hibiscus Sabdariffa* L. Calyx Extracts: In Vitro and in Silico Modelling Approaches,” Evidence‐Based Complementary and Alternative Medicine 2022 (2022): 1–14, 10.1155/2022/1252672. PMC9142284 35646135

[65] S. Haryanti , Y. Widiyastuti , H. Widodo , et al., “Phytochemical Profiling, Antioxidant and Cytotoxic Activity of Turmeric‐ginger Combination Extract Against Cancer Cell Line,” JAPS: Journal of Animal and Plant Sciences 35 (2025): 330–340, 10.36899/japs.2025.2.0027.

[66] D. Tlhapi , N. Malebo , I. T. Manduna , M. Mawunu , and R. C. Chokwe , “Phytochemical Screening and Biological Activities of *Lippia multiflora* Moldenke",” Molecules 30 (2025): 2882, 10.3390/molecules30132882. 40649396 PMC12250754

[67] B. Ngobeni , I. T. Manduna , N. J. Malebo , and S. S. Mashele , “Potential Therapeutic Effects of *Hermannia Depressa* NE Br. Root Extracts,” Journal of Medicinal Plants for Economic Development 8 (2024): 239, 10.4102/jomped.v8i1.239.

[68] N. G. Mariri , N. I. Mongalo , and T. J. Makhafola , “The in Vitro Cytotoxicity, Genotoxicity and LC‐ToF‐MS Profiling of Four South African Plants With Good Antifungal Activity,” South African Journal of Botany 174 (2024): 446–455, 10.1016/j.sajb.2024.09.021.

