# Supplementary material for: Identification of Antioxidant, Antimicrobial, and Cytotoxic Compounds From Hymenocardia acida Tul. Using Ultra‐High‐Performance Liquid Chromatography‐Quadrupole Exactive‐Orbitrap‐Mass Spectrometry and Molecular Networking Approach
Source: Chem Biodivers. 2025 Dec 17;23(2):e02485. doi: 10.1002/cbdv.202502485 (PMC12860518; doi:10.1002/cbdv.202502485)
Supplement: Supplementary file 1 — Supporting File 1: cbdv70802‐sup‐0001‐SuppMat.docx. [file CBDV-23-e02485-s001.docx]

Identification of Antioxidant, Antimicrobial and Cytotoxic Compounds from *Hymenocardia acida* Tul. Using UHPLC-Q Exactive-Orbitrap-MS and Molecular Networking Approach

Ntsoaki Joyce Malebo,^b^ Idah Tichaidza Manduna,^a^ Monizi Mawunu,^c,e^, Ramakwala Christinah Chokwe,^d^ and Dorcas Tlhapi*^,a^

^a^ Centre for Applied Food Sustainability and Biotechnology, Faculty of Health and Environmental Sciences,

Central University of Technology, Bloemfontein 9300, South Africa

^b^ Centre for Innovation in Learning and Teaching, Central University of Technology,

Bloemfontein 9300, South Africa

^c^ Department of Agronomy, Polytechnic Institute, Kimpa Vita University, Luanda P.O. Box 77, Angola

^d^ Department of Chemistry, College of Science Engineering and Technology, University of South Africa, Florida, Johannesburg, 1710, South Africa

^e^ Department of Biology, Faculty of Science and Technology, University of Kinshasa, Kinshasa, P.O. Box 190 Kinshasa XI, Democratic Republic of Congo

***** Correspondence: btlhapi@cut.ac.za

**Network analysis job link**

1. Molecular network ESI(-):ID=b53b092156a64604871064fcba409175

[https://gnps.ucsd.edu/ProteoSAFe/status.jsp?task=](https://gnps.ucsd.edu/ProteoSAFe/status.jsp?task=c4e42d35fcf04dba9ce6fbb77074ec8e) b53b092156a64604871064fcba409175

**
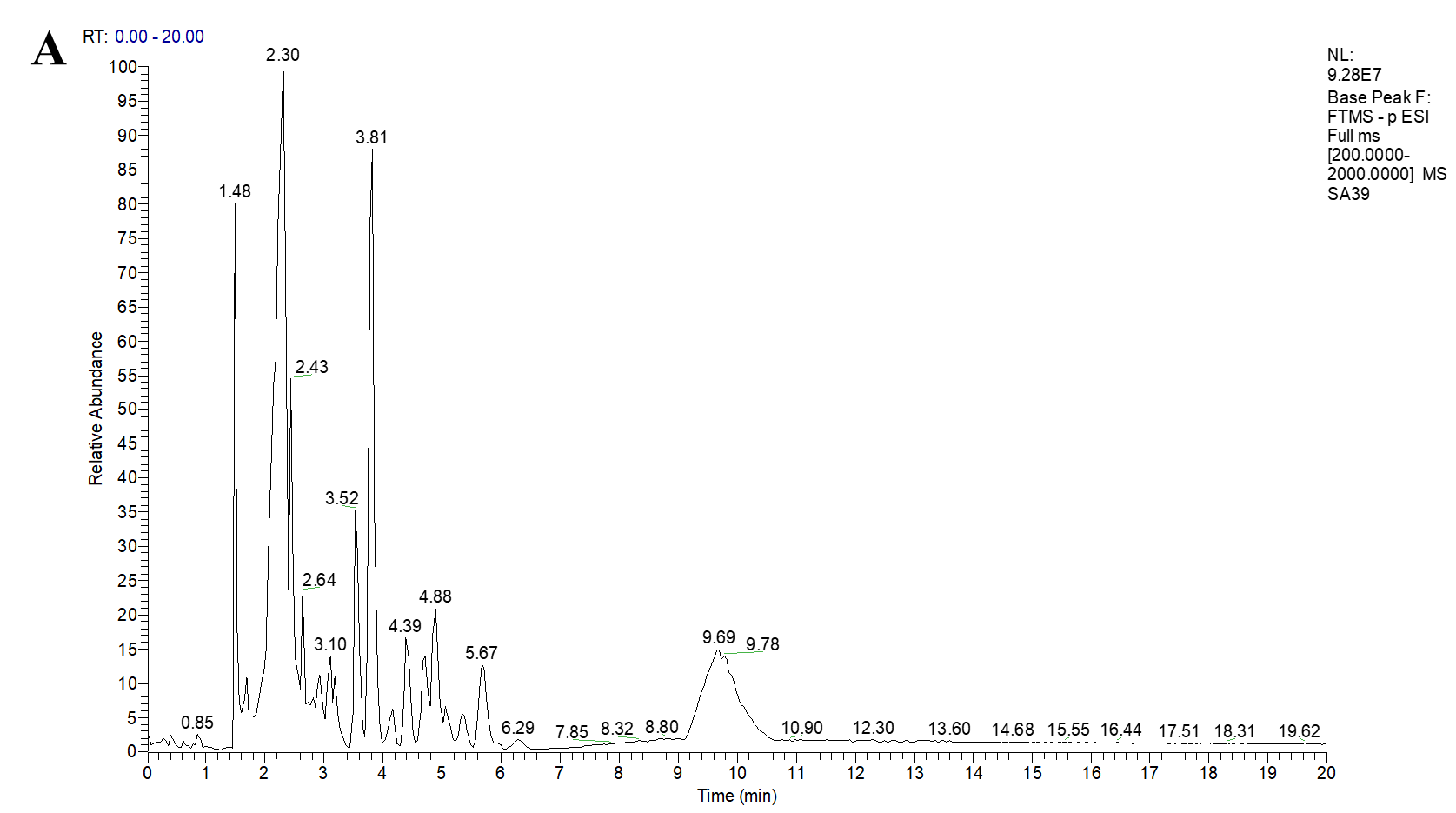
**

**
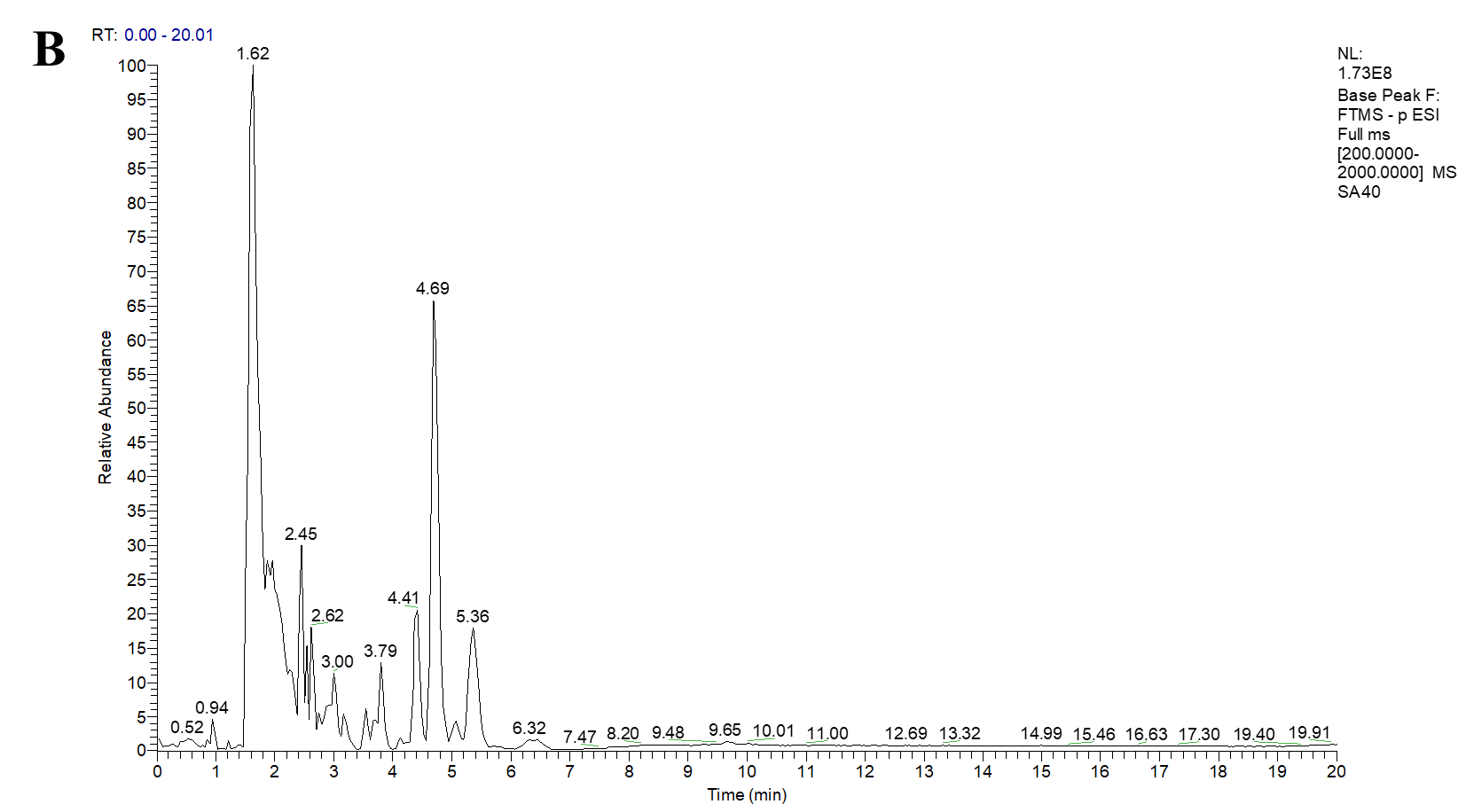
**


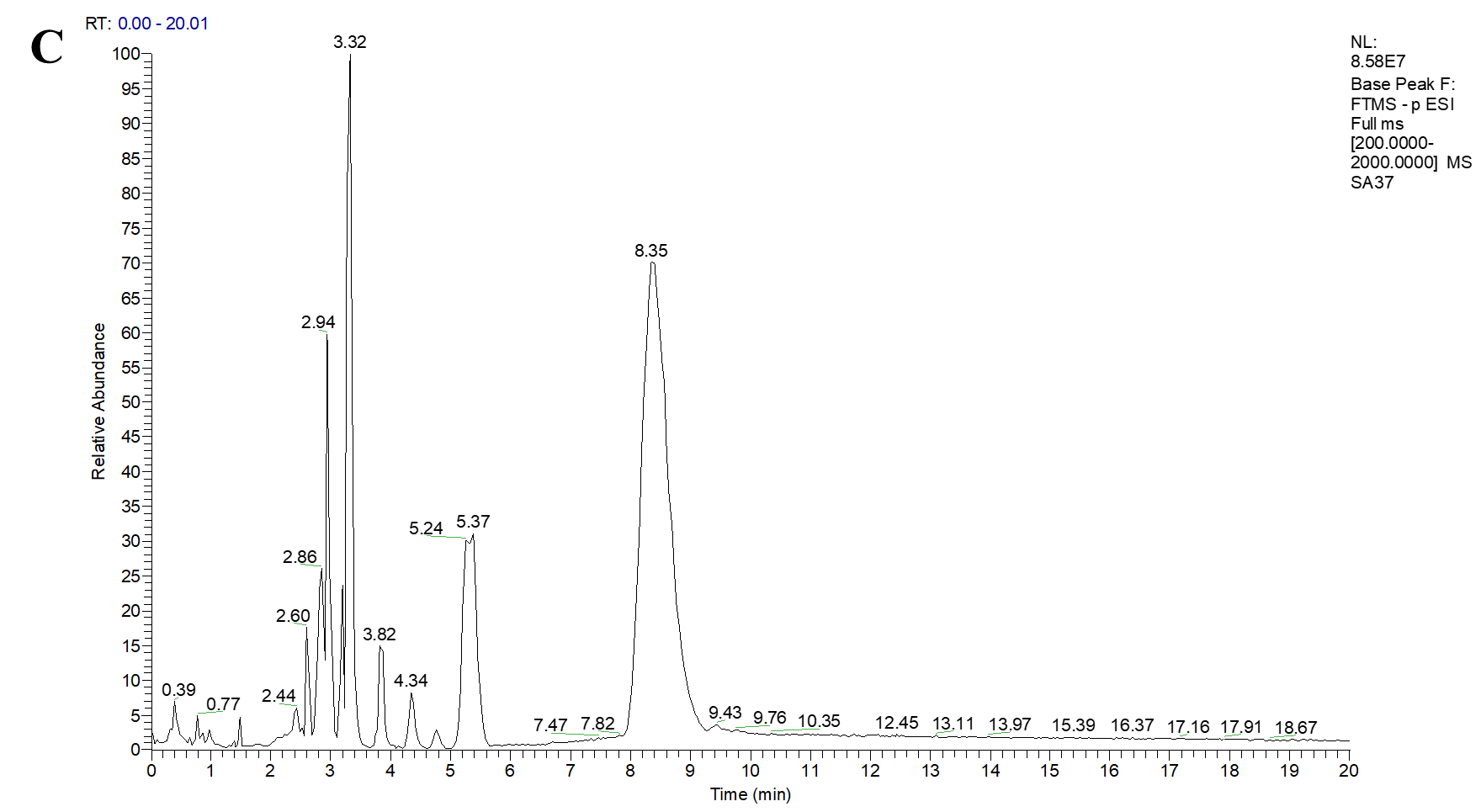


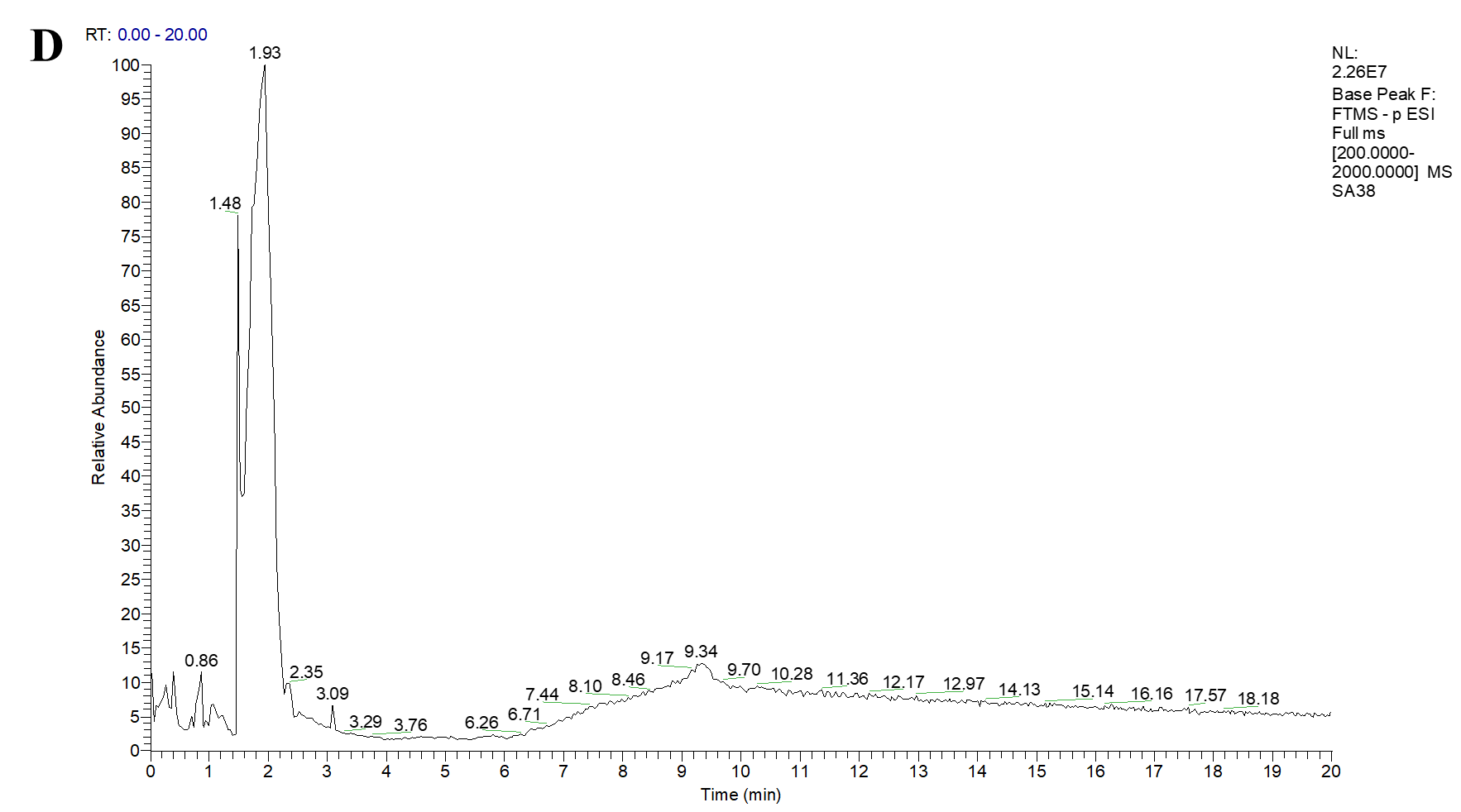


**Figure S1.** UHPLC–Q/Orbitrap/MS HRMS chromatograms with relative abundance and retention time (min) of (**A**) root; (**B**) stem bark; (**C**) dichloromethane leaf; and (**D**) methanol leaf extracts of *Hymenocardia acida* obtained in negative mode electrospray ionization.

**
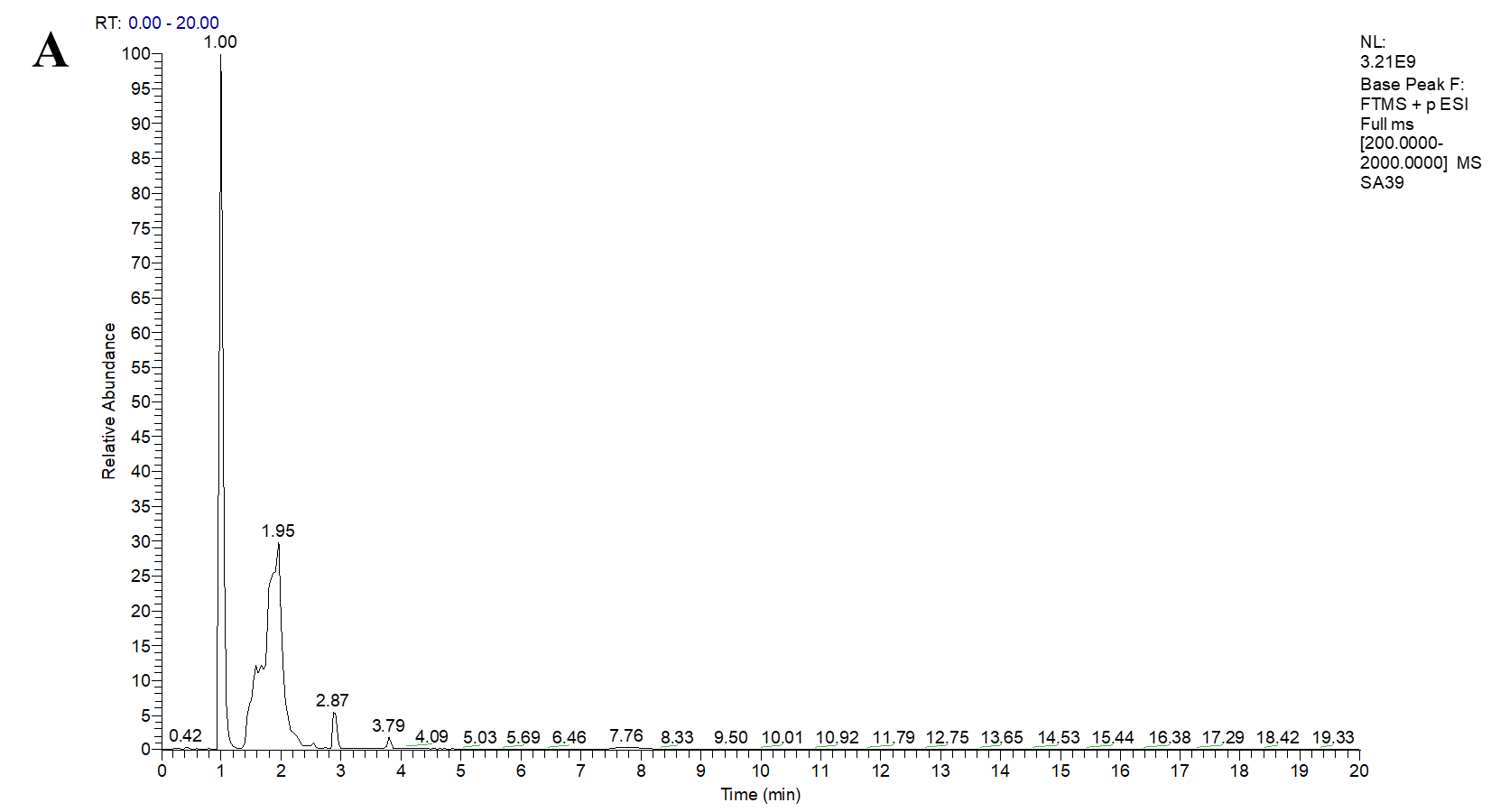
**

**
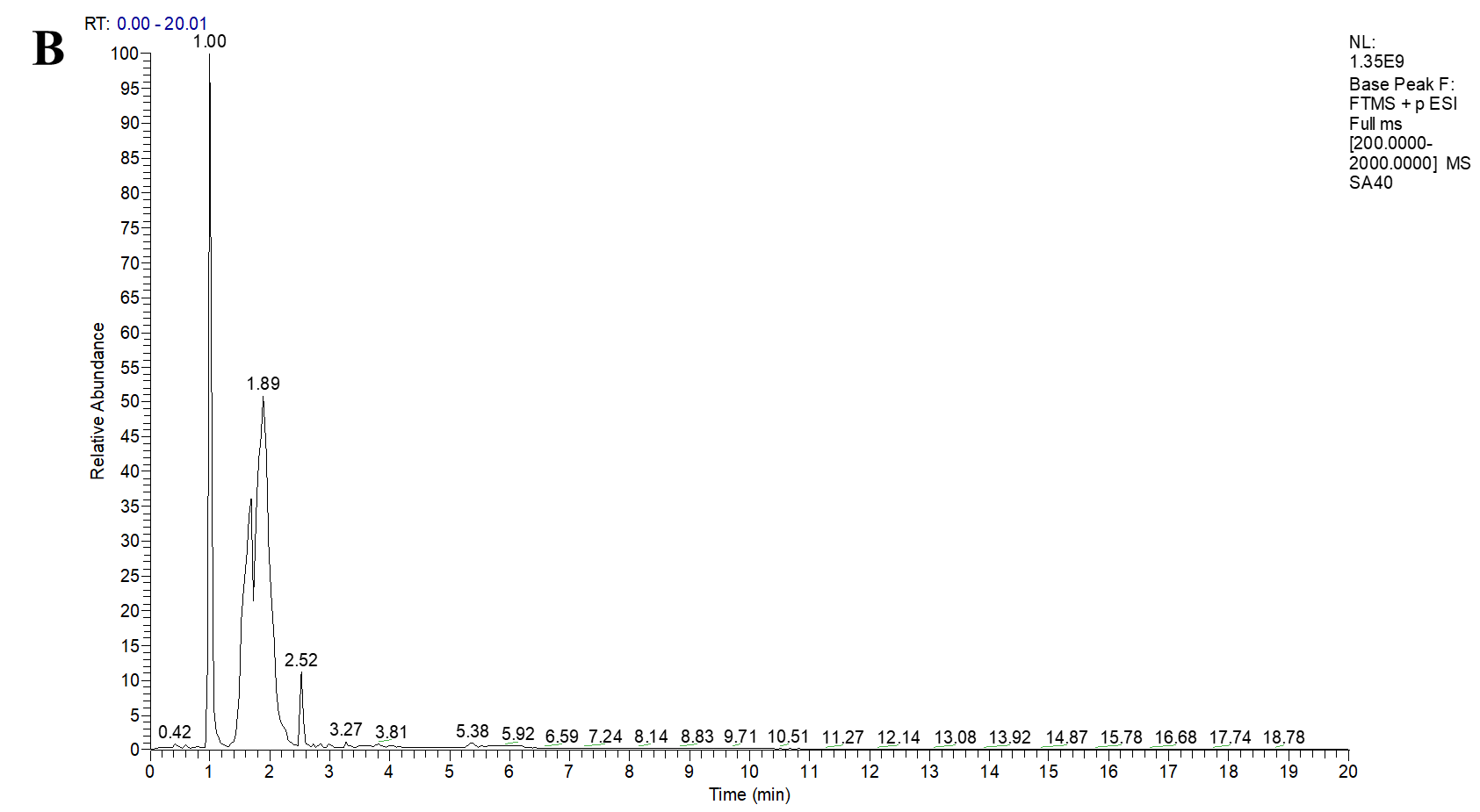
**

**
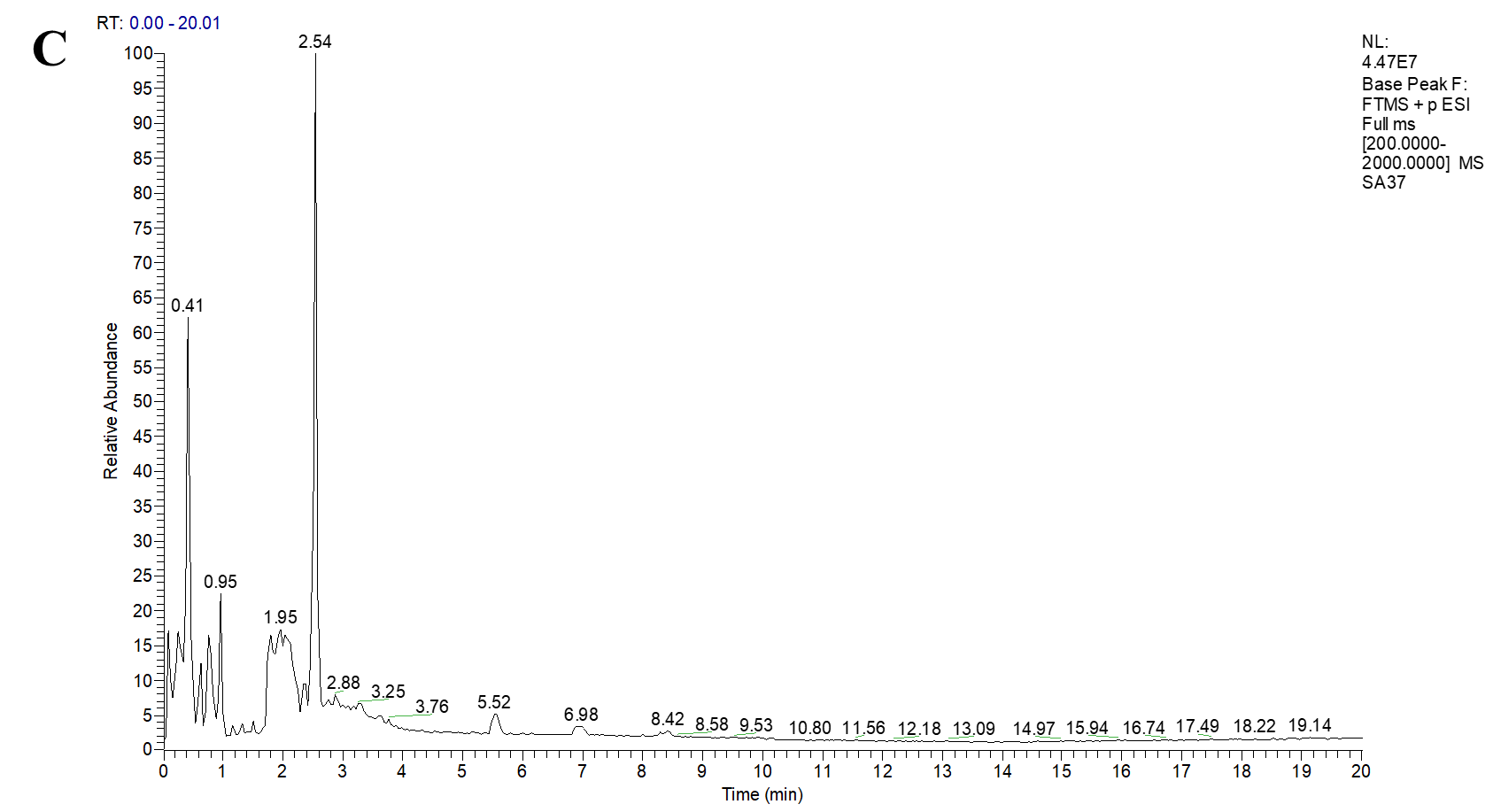
**

**
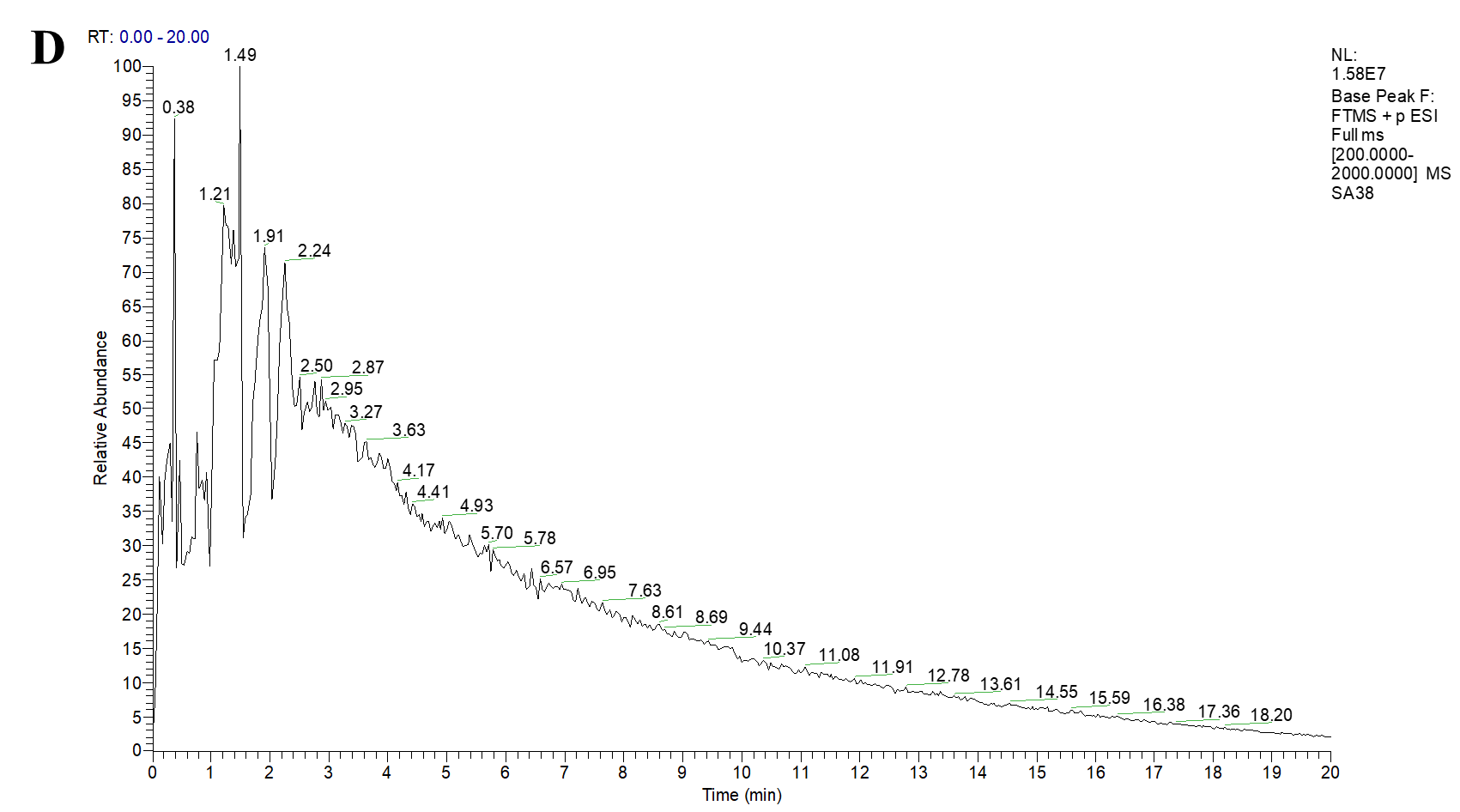
**

**Figure S2.** UHPLC–Q/Orbitrap/MS HRMS chromatograms with relative abundance and retention time (min) of (**A**) root; (**B**) stem bark; (**C**) dichloromethane leaf; and (**D**) methanol leaf extracts of *Hymenocardia acida* obtained in positive mode electrospray ionization.

Metabolite profiling using UHPLC-Q Exactive-Orbitrap-MS

Twenty-six metabolites from different parts of *Hymenocardia acida* were tentatively annotated using UHPLC-Q/Orbitrap/MS analysis. The spectral data of the annotated metabolites was compared with values in the literature. UHPLC-Q/Orbitrap/MS data for the annotated compounds, namely, retention time (Rt), molecular ions [M - H]^-^, MS/MS fragment ions (*m/z*, descriptions in Supplementary Material), and main product ions, were provided in Table **1**. The retention times of the metabolites were compared with the standard reference data to detect the metabolites in the *H. acida* extracts. Both electrospray ionization modes (ESI (-/+); Figure S1 and S2, Supplementary Material) were generated using UHPLC-Q/Orbitrap/MS. However, the negative ionization mode was chosen for further sample analysis because it produces a greater abundance of ions and provides information-rich spectra. Various classes of secondary metabolites have been annotated, including flavonoids, fatty acids, coumarins, terpenoids, alkaloids, anthocyanins, saponins, polyphenols, and glycosides.

Compound **1** was annotated with an [M-H]^-^ ion at *m/z* 339.20 (Figure S3, Supplementary Material) as 8-prenylnaringenin (C_20_H_20_O_5_).^20^ Compounds **2** (ion at *m/z*: 389.17, C_19_H_18_O_9_) and **3** (ion at *m/z*: 339.94, C_22_H_44_O_2_) were annotated as scaposin and behenic acid, respectively (Figures S4 and S5, respectively, in the Supplementary Material). These compounds were previously reported by Khurm et al. (2023) and Isaac et al. (2014), respectively.^21,22^ Compound **4**, with a precursor ion at *m/z* 339.07 [M - H]^-^, was tentatively annotated as 6,7-dihydroxycoumarin-6-glucoside (Esculin, Figure S6, Supplementary Material).^23^ Compound **5**, with a [M - H]^-^ ion at *m/z* 483.12 (Figure S7, Supplementary Material), was tentatively annotated as poricoic acid B with a molecular formula of C_30_H_44_O_5_,^24^ whereas compound **6**, with a [M - H]^-^ ion at *m/z* 427.18 (Figure S8, Supplementary Material), was identified as isomajdine with a molecular formula of C_23_H_28_N_2_O_6_.^25^ Compounds **7** and **8** showed the presence of yohimbic acid (*m/z* 339.12, Figure S9, Supplementary Material) and 7-hydroxy-3-methylflavone (*m/z* 251.03, Figure S10, Supplementary Material).^26,27^ Furthermore, compounds **9** (Rt = 1.76 min), **10** (Rt = 1.68 min), **11** (Rt = 1.68 min), and **12** (Rt = 0.48 min), with [M - H]^-^ ions at *m/z* 596.16 (Figure S11, Supplementary Material), *m/z* 595.15 (Figure S12, Supplementary Material), *m/z* 595.15 (Figure S13, Supplementary Material), and *m/z* 595.27 (Figure S14, Supplementary Material), respectively were confirmed as delphinidin-3-*O*-sambubioside ([C_26_H_29_O_16_]^+^), quercetin-3-*O*-vicianoside (C_26_H_28_O_16_), eriodictyol-7-*O*-neohesperidoside (Neoeriocitrin) (C_27_H_32_O_15_) and quercetin-3-arabinoglucoside (Peltatoside) (C_26_H_28_O_16_), respectively.^28-31^ Compounds **13** (*m/z* 919.50 [M - H]^-^, Figure S15, Supplementary Material), **14** (*m/z* 1083.25 [M - H]^-^, Figure S16, Supplementary Material), **15** (*m/z* 467.16 [M - H]^-^, Figure S17, Supplementary Material), and **16** (*m/z* 295.23 [M - H]^-^, Figure S18, Supplementary Material) were annotated as furostane base -1H2O + 1O, *O*-Hex, *O*-Hex-Hex (C_45_H_74_O_19_), furostane base + *O*-Hex, *O*-Hex-Hex-Hex (C_51_H_86_O_24_), dehydroeburicoic acid (C_31_H_48_O_3_), and esmolol (C_16_H_25_NO_4_), respectively (MassBank-RIKEN-PR308985; MassBank-RIKEN-PR310726).^32,33^ However, 3',4'-dimethoxy-7-hydroxyflavone (**17**), 6-gingerol (**18**), medicagenic acid (**19**), 6-paradol (**20**), quercitrin (**21**), and 3-glucosyl-2,3′,4,4′,6- pentahydroxybenzophenone (**22**) with precursor ions at *m/z* 297.24 [M - H]^-^, *m/z* 293.18 [M - H]^-^, *m/z* 501.11 [M - H]^-^, *m/z* 277.20[M - H]^-^, *m/z* 447.09 [M - H]^-^ and *m/z* 423.17 [M - H]^-^, respectively were annotated at 2.88 min, 1.46 min, 1.55 min, 2.33 min, 1.97 min, and 1.91 min, respectively, as shown in Figures S19 and S20–S24, respectively in the Supplementary Material) (MassBank-BS-BS003750).^33,34,33,35,33^ Compounds **23** (*m/z* 533.38 [M - H]^-^, Figure S25, Supplementary Material), **24** (*m/z* 307.19 [M - H]^-^, Figure S26, Supplementary Material), **25** (*m/z* 525.30 [M - H]^-^, Figure S27, Supplementary Material), and **26** (*m/z* 283.03 [M - H]^-^, Figure S28, Supplementary Material) were identified as 6ʹʹ -*O*-malonylgenistin (C_24_H_22_O_13_), eicosadieneoic acid (C_20_H_36_O_2_), paeoniflorin (C_23_H_28_O_11_), and stearic acid (C_18_H_36_O_2_), respectively (MSBNK-Antwerp_Univ METOX_N109326_B8BB).^36-38^

**
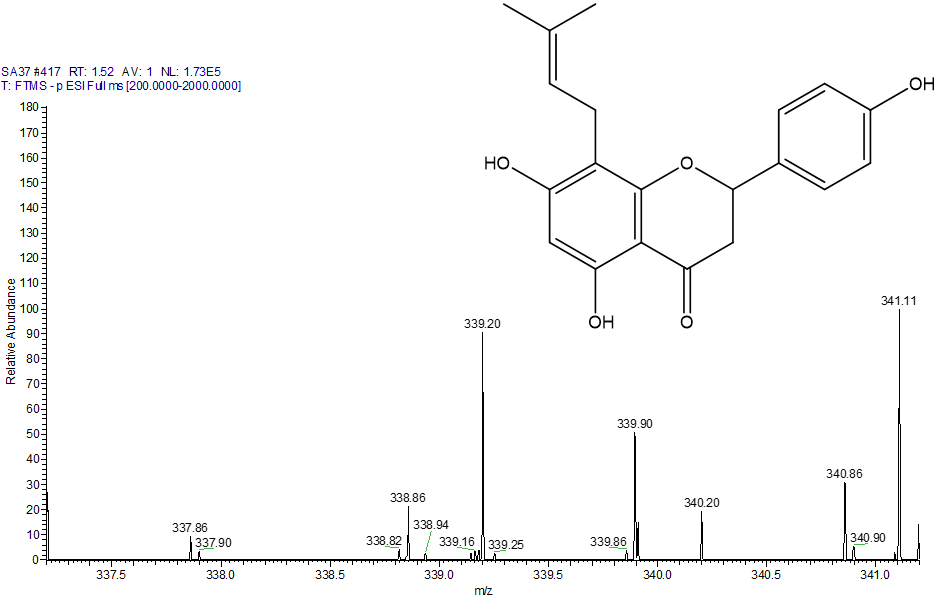
**

**Figure S3.** UHPLC–Q/Orbitrap/MS HRMS spectrum showing fragments of 8-prenylnaringenin.

**
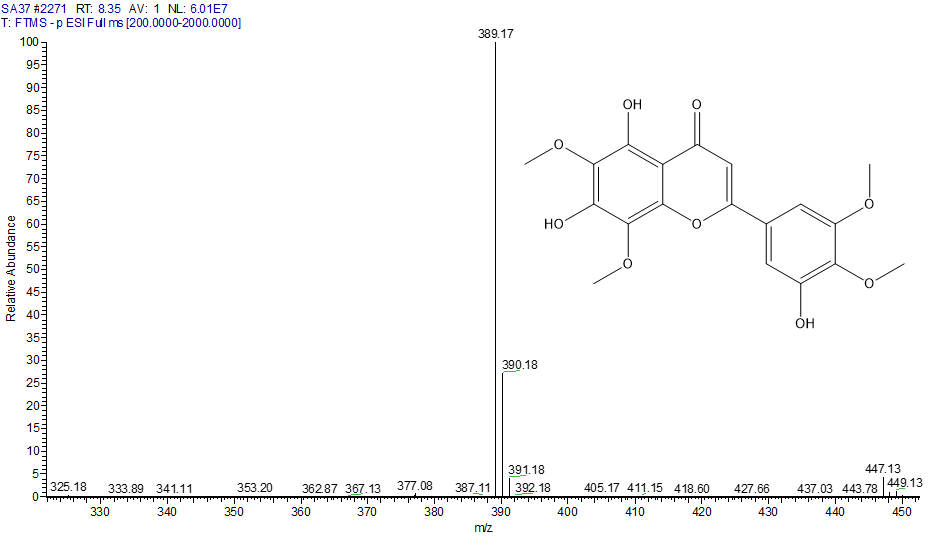
**

**Figure S4.** UHPLC–Q/Orbitrap/MS HRMS spectrum showing fragments of scaposin.

**
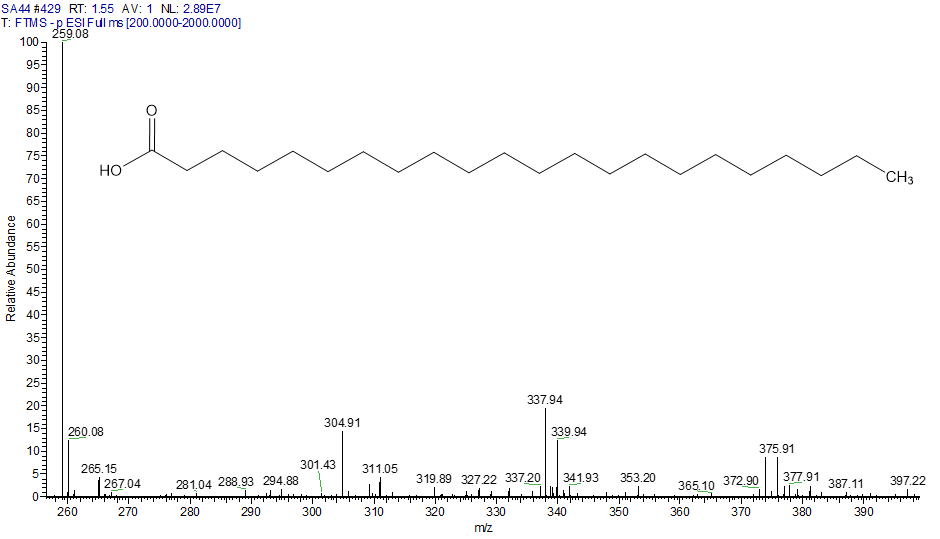
**

**Figure S5.** UHPLC–Q/Orbitrap/MS HRMS spectrum showing fragments of behenic acid.

**
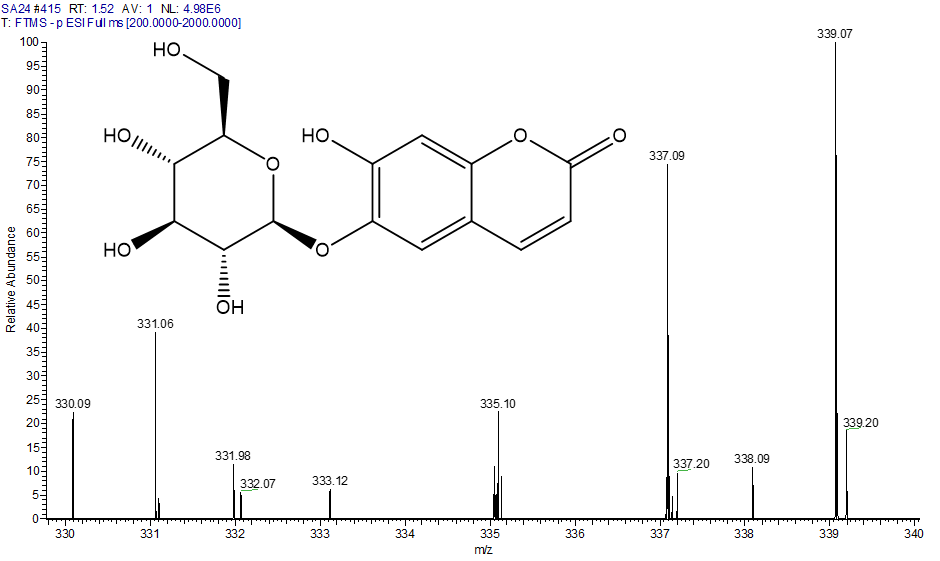
**

**Figure S6.** UHPLC–Q/Orbitrap/MS HRMS spectrum showing fragments of 6,7-dihydroxycoumarin-6-glucoside (Esculin).

**
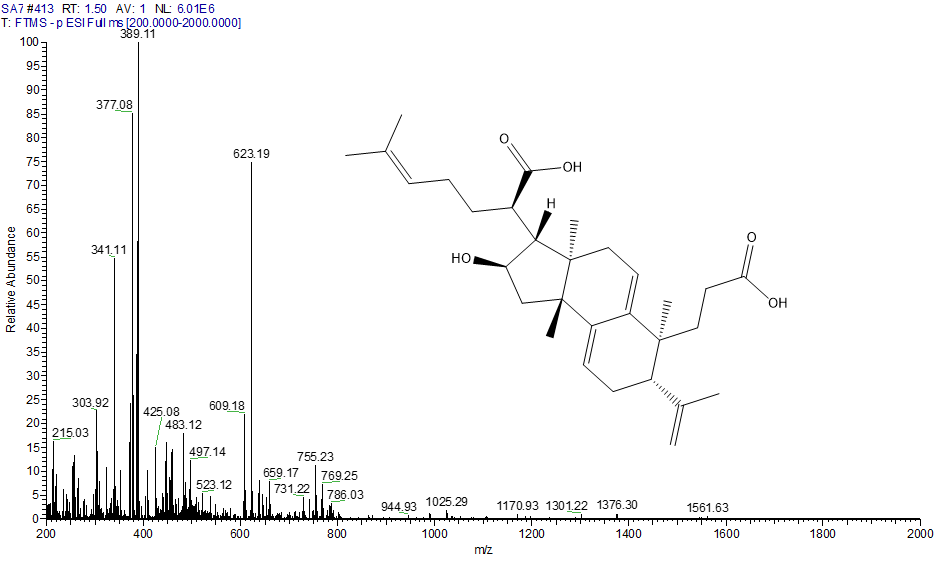
**

**Figure S7.** UHPLC–Q/Orbitrap/MS HRMS spectrum showing fragments of poricoic acid B.

**
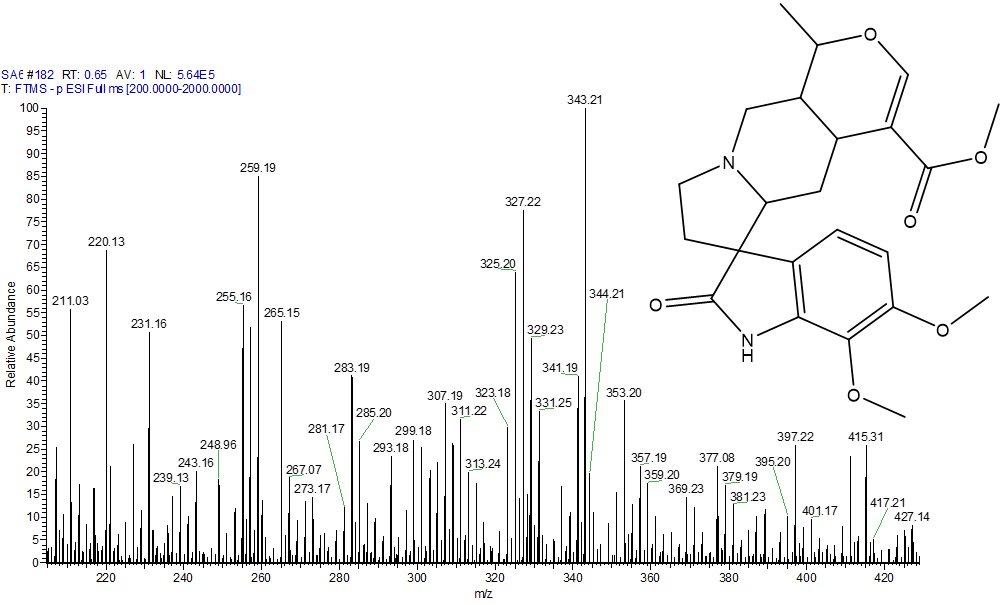
**

**Figure S8.** UHPLC–Q/Orbitrap/MS HRMS spectrum showing fragments of isomajdine.

**
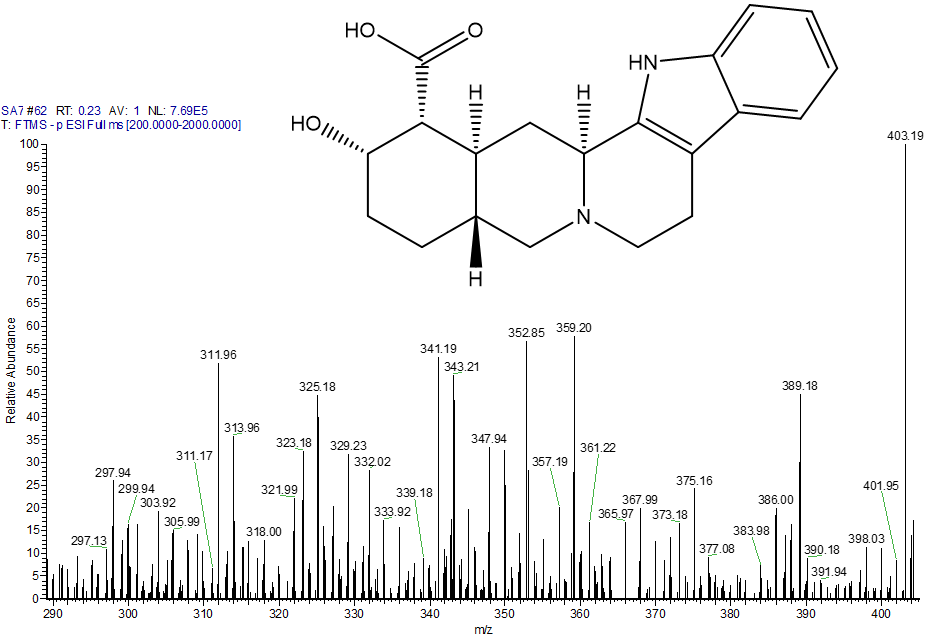
**

**Figure S9.** UHPLC–Q/Orbitrap/MS HRMS spectrum showing fragments of yohimbic acid.

**
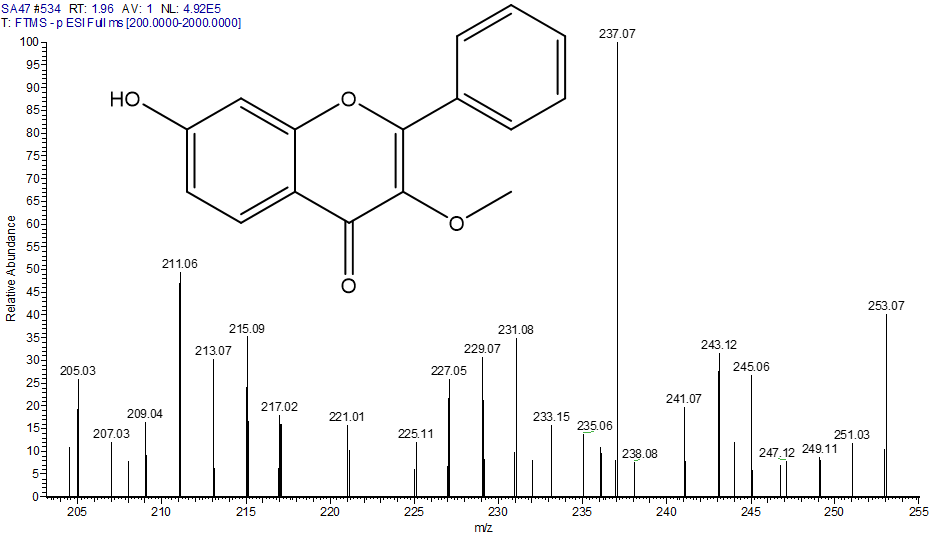
**

**Figure S10.** UHPLC–Q/Orbitrap/MS HRMS spectrum showing fragments of 7-hydroxy-3-methylflavone.

**
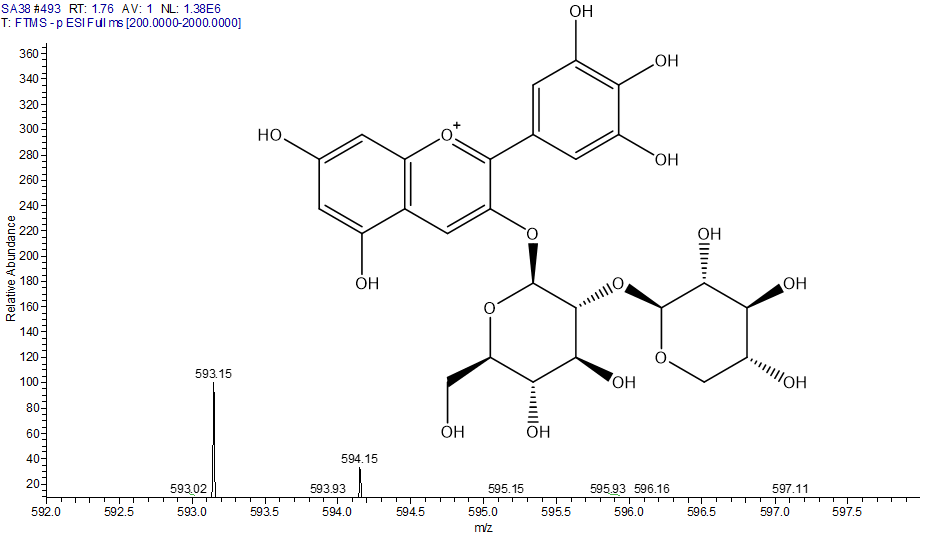
**

**Figure S11.** UHPLC–Q/Orbitrap/MS HRMS spectrum showing fragments of delphinidin-3-*O*-sambubioside.

**
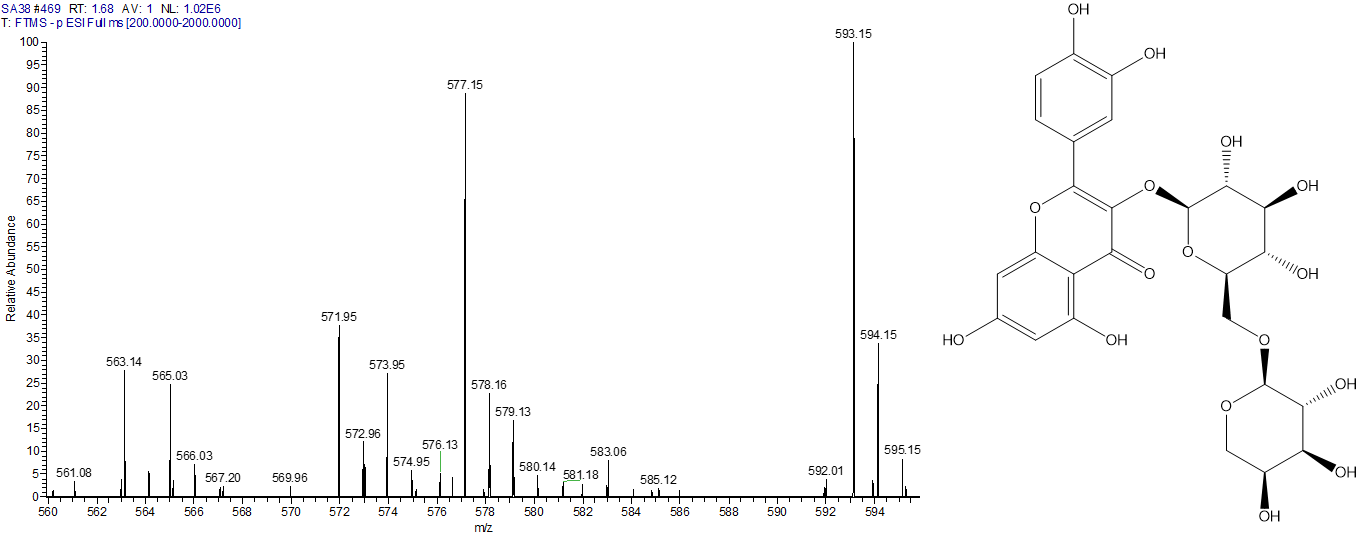
**

**Figure S12.** UHPLC–Q/Orbitrap/MS HRMS spectrum showing fragments of quercetin-3-*O*-vicianoside.

**
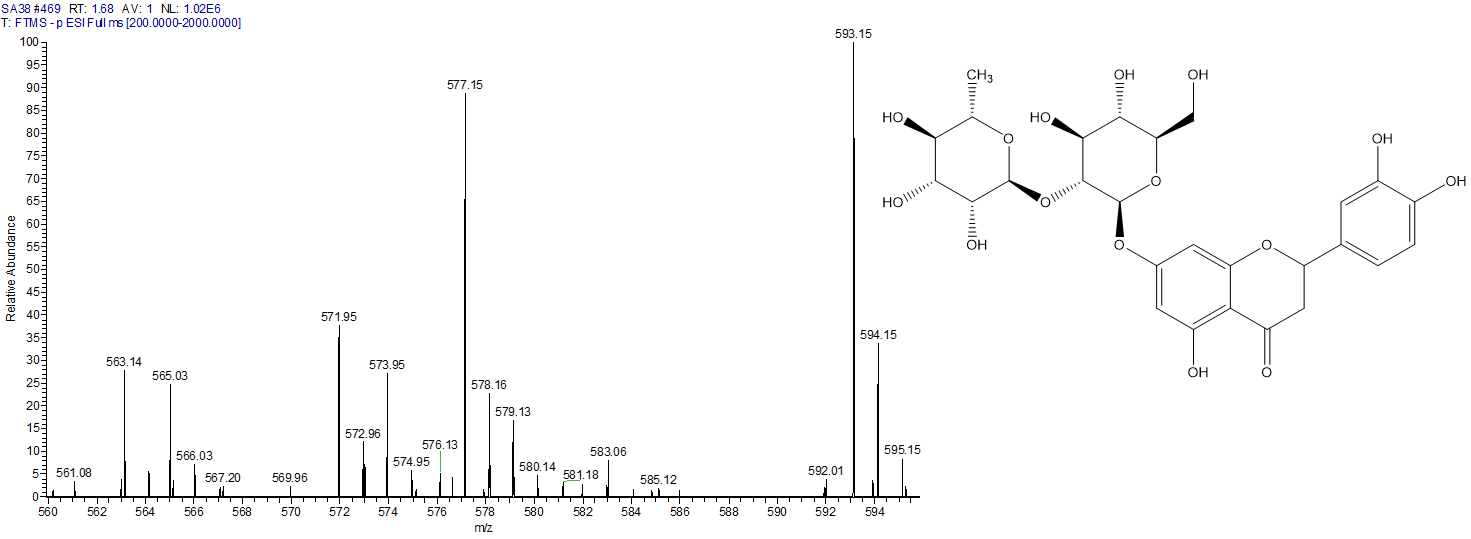
Figure S13.** UHPLC–Q/Orbitrap/MS HRMS spectrum showing fragments of eriodictyol-7-*O*-neohesperidoside (Neoeriocitrin).

**
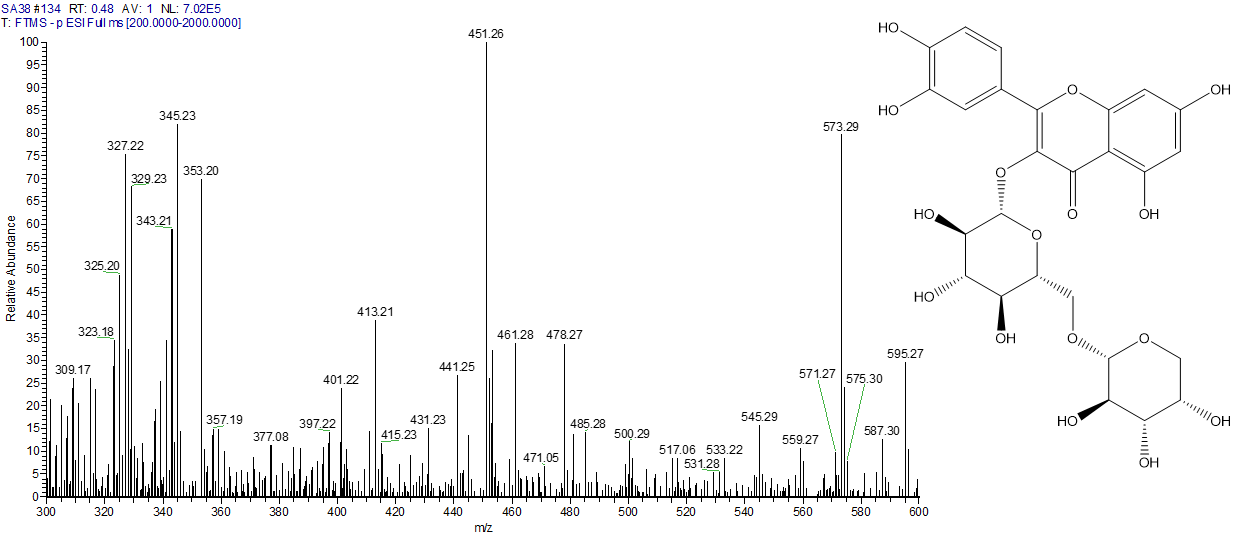
**

**Figure S14.** UHPLC–Q/Orbitrap/MS HRMS spectrum showing fragments of quercetin-3-arabinoglucoside (Peltatoside).

**
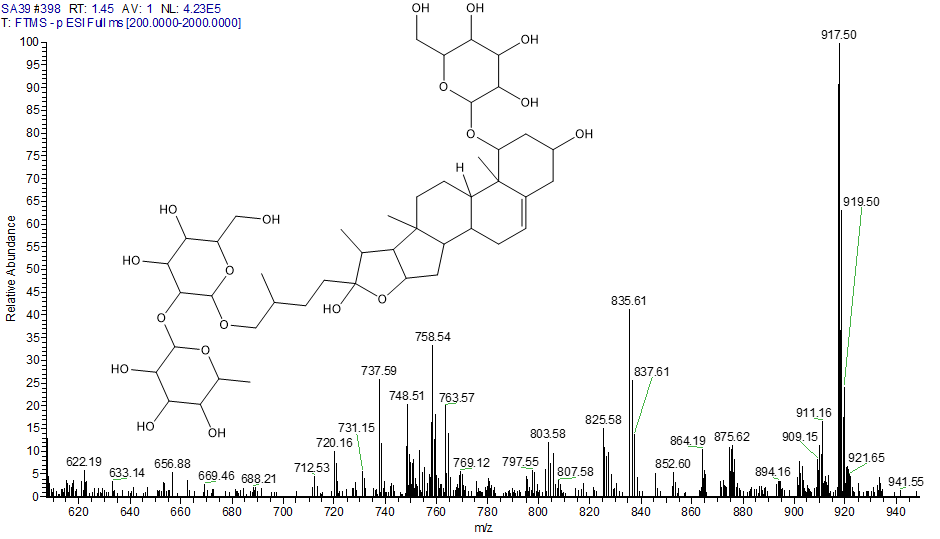
**

**Figure S15.** UHPLC–Q/Orbitrap/MS HRMS spectrum showing fragments of furostane base -2H + 1O, O-Hex, O-Hex-dHex.

**
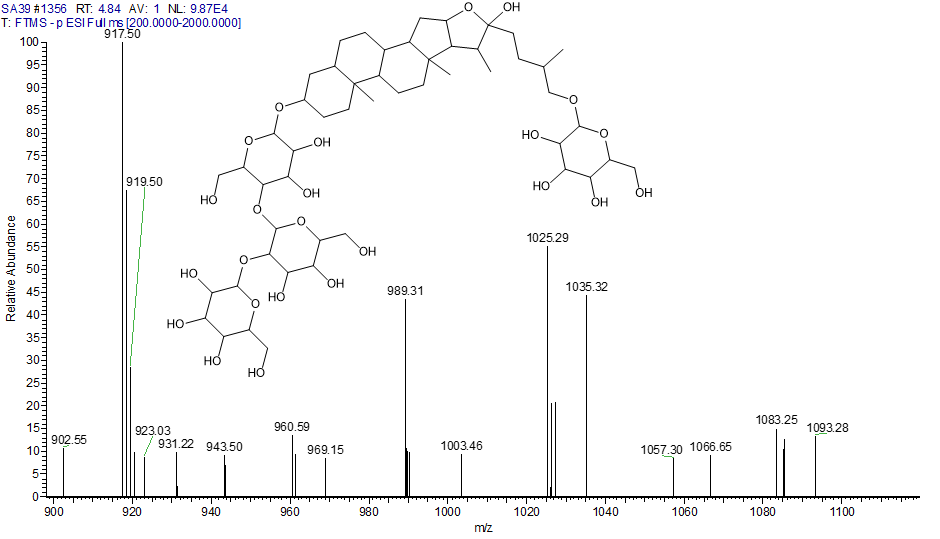
**

**Figure S16.** UHPLC–Q/Orbitrap/MS HRMS spectrum showing fragments of furostane base + O-Hex, O-Hex-Hex-Hex.

**
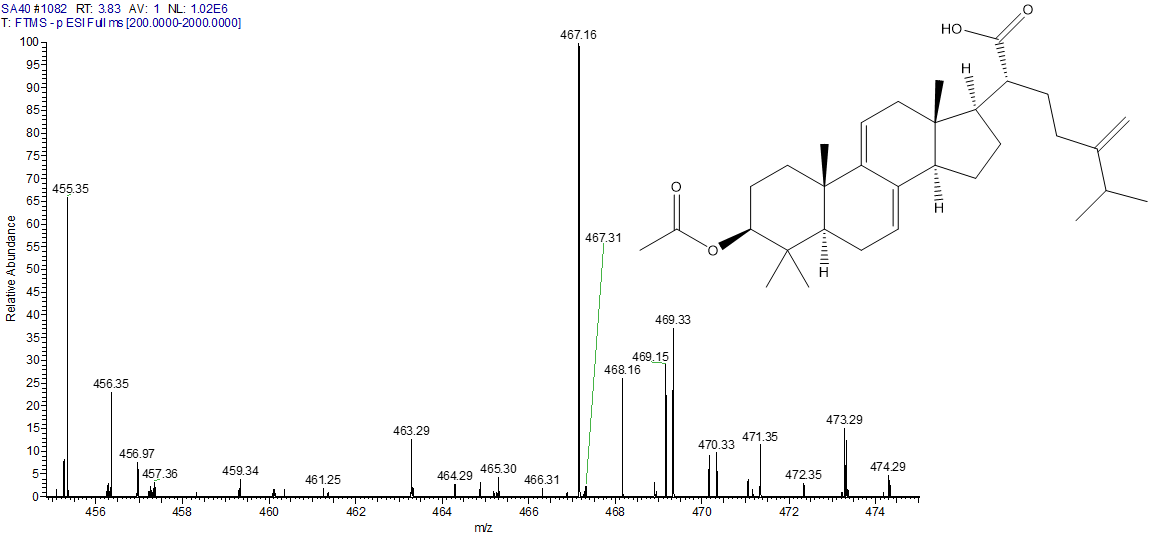
**

**Figure S17.** UHPLC–Q/Orbitrap/MS HRMS spectrum showing fragments of dehydroeburicoic acid.

**
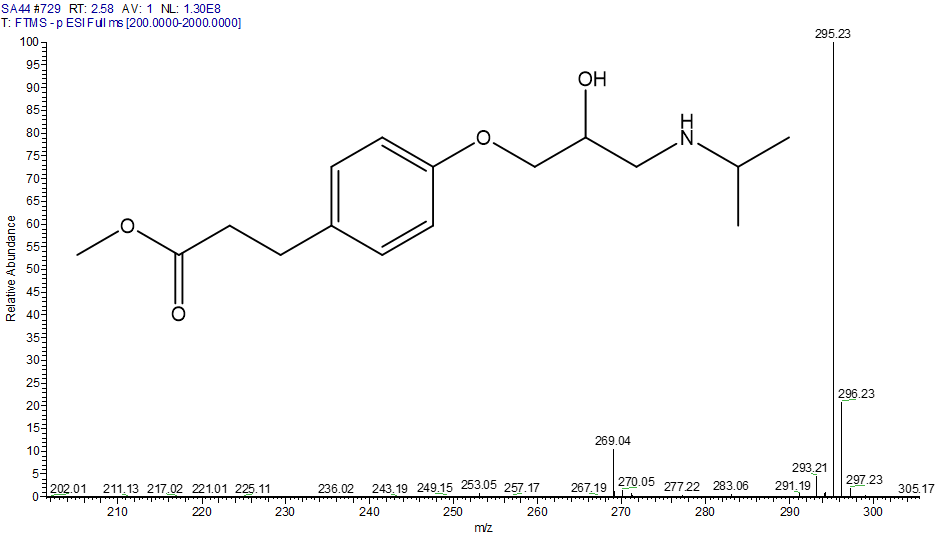
**

**Figure S18.** UHPLC–Q/Orbitrap/MS HRMS spectrum showing fragments of esmolol.

**
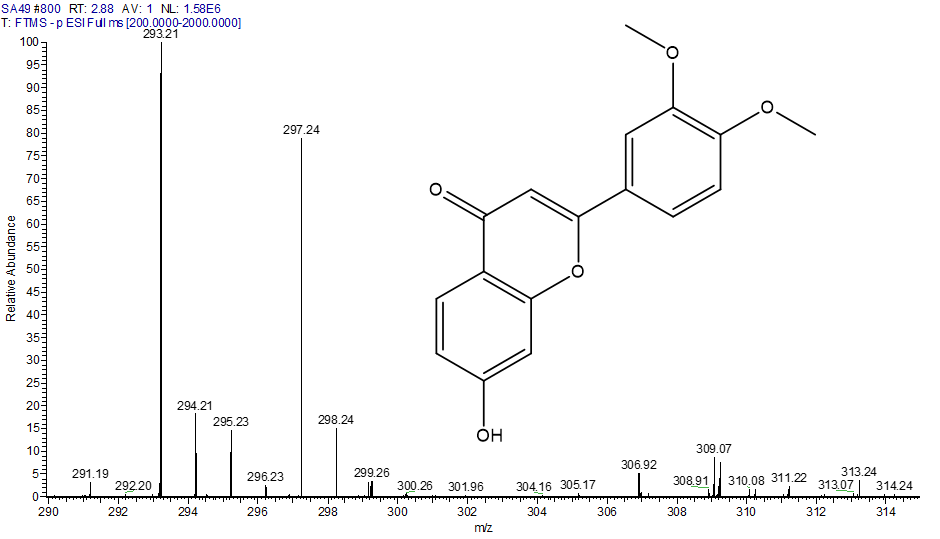
**

**Figure S19.** UHPLC–Q/Orbitrap/MS HRMS spectrum showing fragments of 3',4'-dimethoxy-7-hydroxyflavone.

**
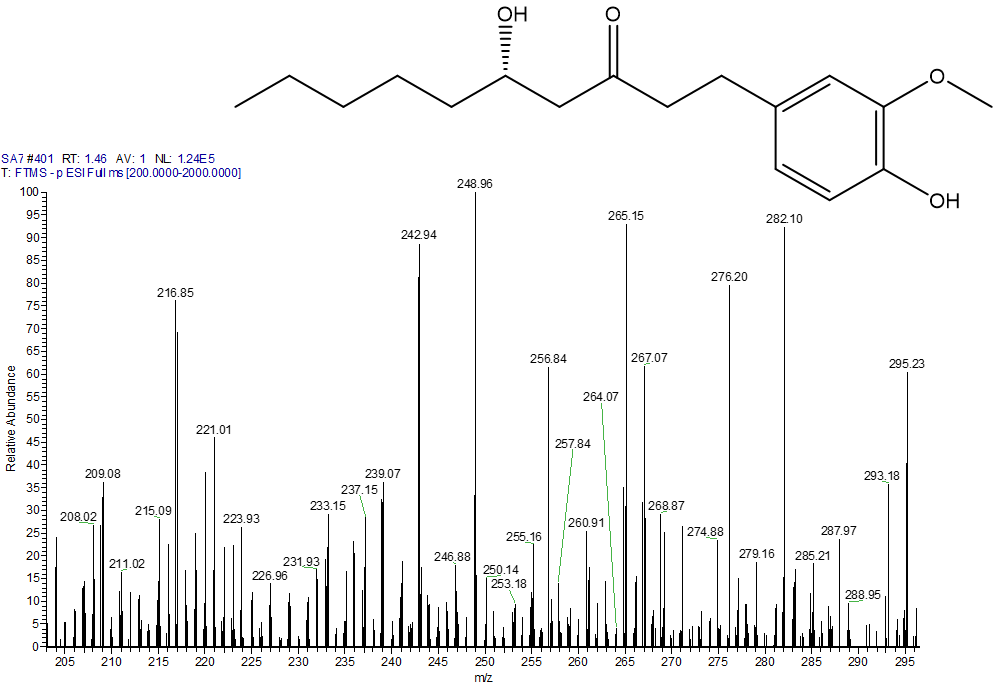
**

**Figure S20.** UHPLC–Q/Orbitrap/MS HRMS spectrum showing fragments of 6-gingerol.

**
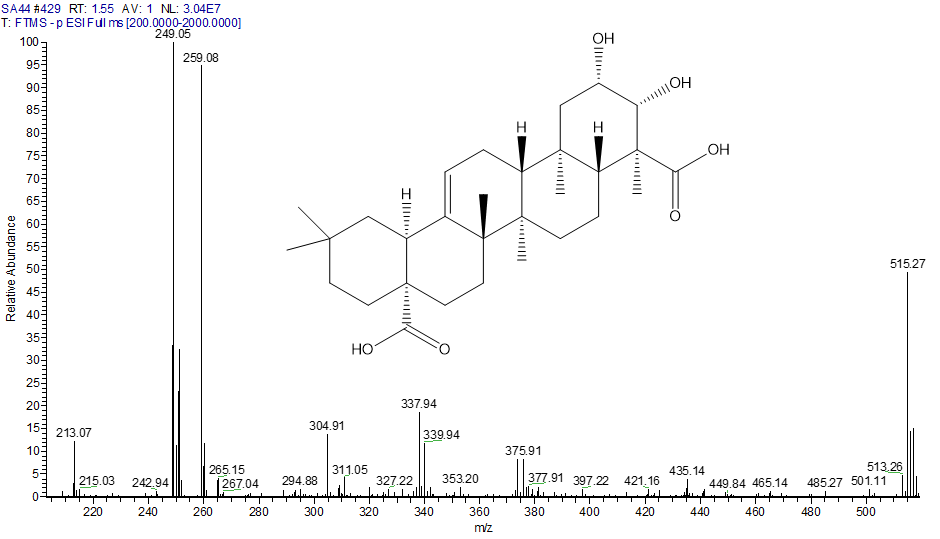
**

**Figure S21.** UHPLC–Q/Orbitrap/MS HRMS spectrum showing fragments of medicagenic acid.

**
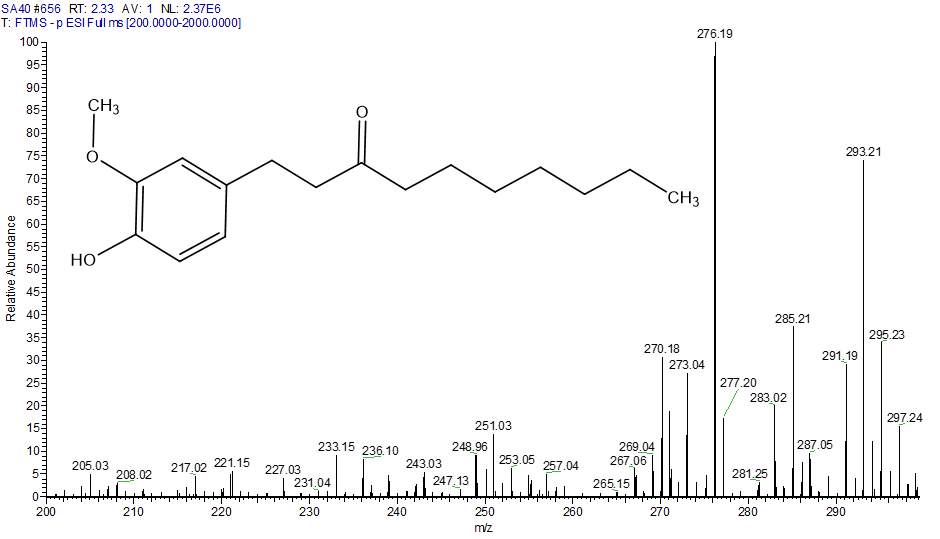
**

**Figure S22.** UHPLC–Q/Orbitrap/MS HRMS spectrum showing fragments of 6-paradol.

**
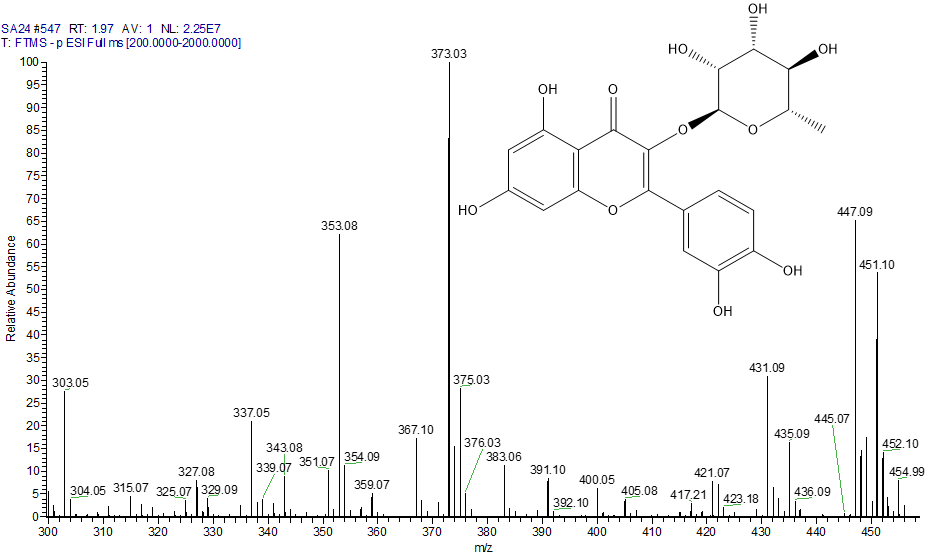
**

**Figure S23.** UHPLC–Q/Orbitrap/MS HRMS spectrum showing fragments of quercitrin.

**
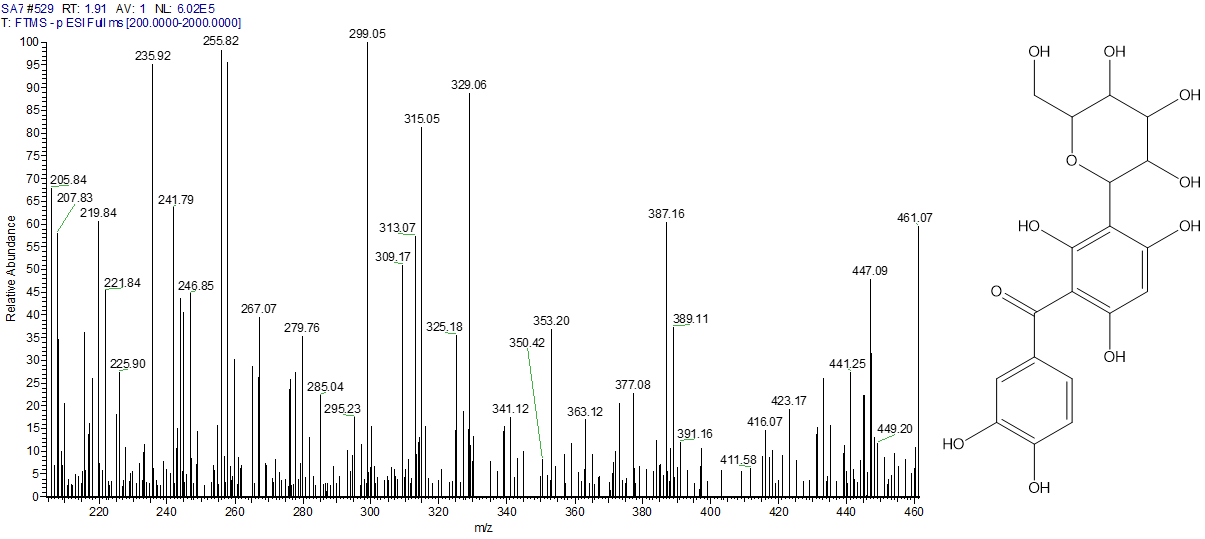
**

**Figure S24.** UHPLC–Q/Orbitrap/MS HRMS spectrum showing fragments of 3-glucosyl-2,3′,4,4′,6- pentahydroxybenzophenone.

**
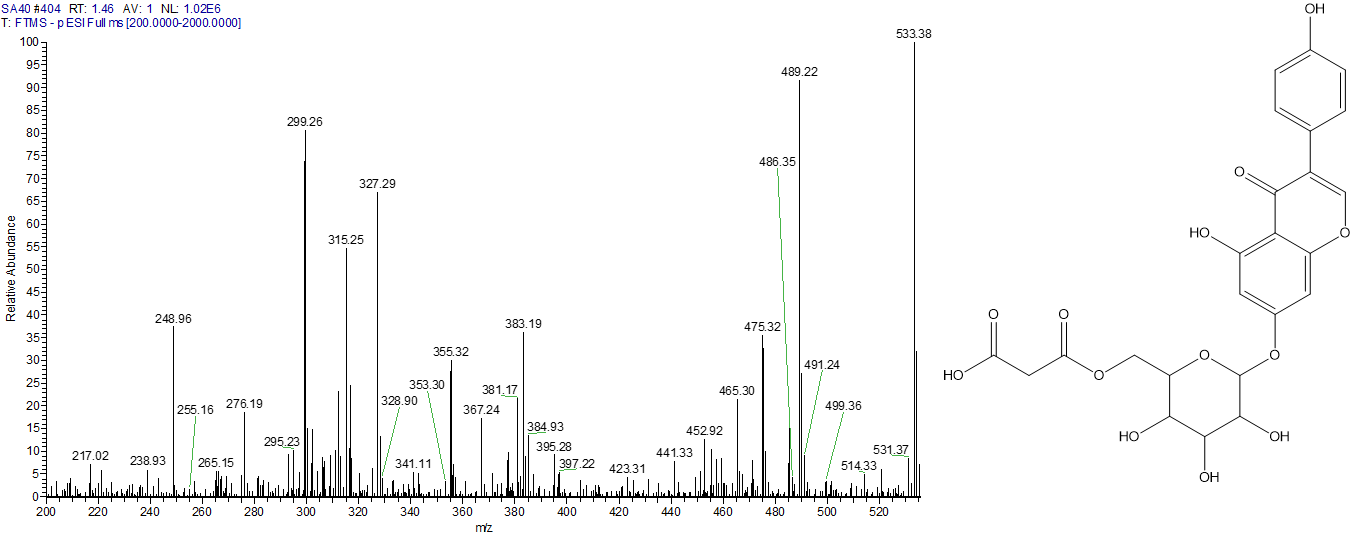
**

**Figure S25.** UHPLC–Q/Orbitrap/MS HRMS spectrum showing fragments of 6ʹʹ -*O*-malonylgenistin.

**
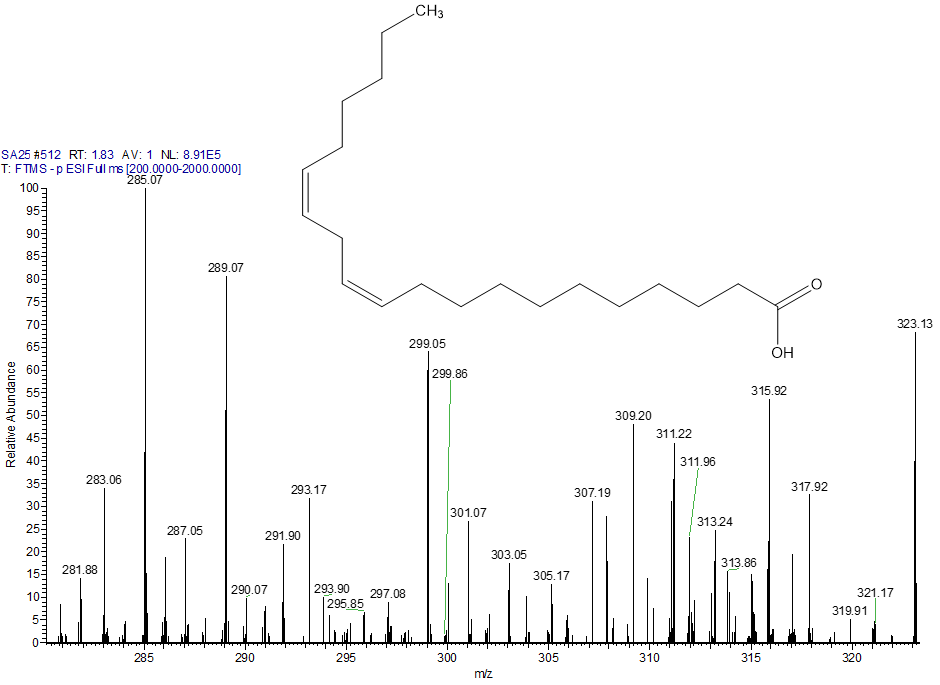
**

**Figure S26.** UHPLC–Q/Orbitrap/MS HRMS spectrum showing fragments of eicosadieneoic acid.

**
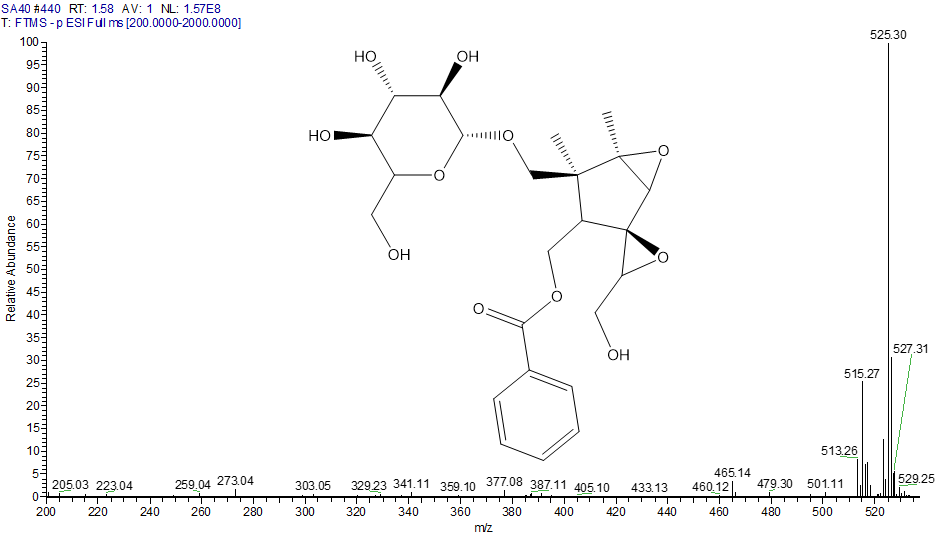
**

**Figure S27.** UHPLC–Q/Orbitrap/MS HRMS spectrum showing fragments of paeoniflorin.

**
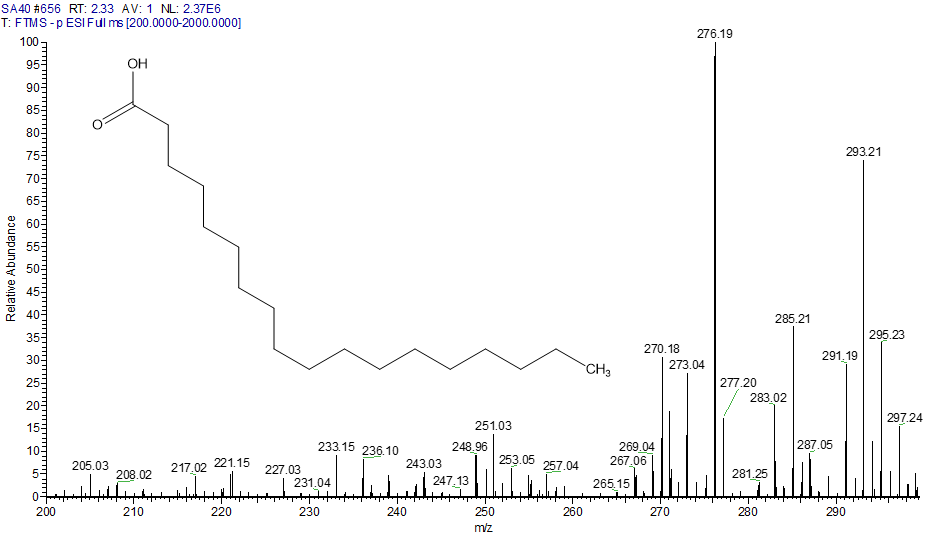
**

**Figure S28.** UHPLC–Q/Orbitrap/MS HRMS spectrum showing fragments of stearic acid.
